# Potent effects of dioscin against liver fibrosis

**DOI:** 10.1038/srep09713

**Published:** 2015-04-08

**Authors:** Xiaoling Zhang, Xu Han, Lianhong Yin, Lina Xu, Yan Qi, Youwei Xu, Huijun Sun, Yuan Lin, Kexin Liu, Jinyong Peng

**Affiliations:** 1 College of Pharmacy, Dalian Medical University, Western 9 Lvshunnan Road, Dalian 116044, China

## Abstract

We previously reported the promising effects of dioscin against liver injury, but its effect on liver fibrosis remains unknown. The present work investigated the activities of dioscin against liver fibrosis and the underlying molecular mechanisms. Dioscin effectively inhibited the cell viabilities of HSC-T6, LX-2 and primary rat hepatic stellate cells (HSCs), but not hepatocytes. Furthermore, dioscin markedly increased peroxisome proliferator activated receptor-γ (PPAR-γ) expression and significantly reduced a-smooth muscle actin (α-SMA), transforming growth factor-β1 (TGF-β1), collagen α1 (I) (COL1A1) and collagen α1 (III) (COL3A1) levels *in vitro*. Notably, dioscin inhibited HSCs activation and induced apoptosis in activated HSCs. *In vivo*, dioscin significantly improved body weight and hydroxylproline, laminin, α-SMA, TGF-β1, COL1A1 and COL3A1 levels, which were confirmed by histopathological assays. Dioscin facilitated matrix degradation, and exhibited hepatoprotective effects through the attenuation of oxidative stress and inflammation, in addition to exerting anti-fibrotic effects through the modulation of the TGF-β1/Smad, Wnt/β-catenin, mitogen-activated protein kinase (MAPK) and mitochondrial signaling pathways, which triggered the senescence of activated HSCs. In conclusion, dioscin exhibited potent effects against liver fibrosis through the modulation of multiple targets and signaling pathways and should be developed as a novel candidate for the treatment of liver fibrosis in the future.

Hepatic fibrosis is a common consequence of chronic liver injury caused by a variety of etiological factors. Hepatic fibrosis leads to cirrhosis^1^, which is accompanied by significant morbidity and mortality and leads to the distortion of normal architecture and nodule formation, hepatocellular carcinoma, and ultimately liver failure^2^. Fibrotic diseases account for up to 45% of deaths in the developed countries. However, effective anti-fibrotic therapies are lacking^3^.

Many biological processes and signaling pathways can affect and modulate hepatic fibrosis. The pathological process of hepatic fibrosis is characterized by the excessive production of extracellular matrix (ECM) proteins^4^, and the activation of hepatic stellate cells (HSCs) plays a critical role in the development of hepatic fibrosis^1^. HSCs undergo obvious phenotypic alterations in response to liver damage, transitioning from a quiescent stage to an activated stage that includes increased cell proliferation, α-SMA expression and ECM production^5^. Collagen generation and degradation mediated by matrix metalloproteinases (MMPs) and tissue inhibitors of metalloproteinases (TIMPs) can affect the outcome of fibrosis. Therefore, the balance between MMPs and TIMPs is crucial in collagen degradation^6^.

The mechanisms of liver fibrosis have been widely investigated. Oxidative stress is likely an important phenomenon that may modulate inflammatory and fibrotic responses^7^. Additionally, chronic inflammatory reaction also plays an important role in triggering liver fibrosis^8^. Other signaling pathways, including TGF-β/Smad, Wnt/β- catenin, MAPKs, cellular senescence and apoptosis, are all associated with liver fibrosis^9^,^10^,^11^,^12^. TGF-β is widely described as the most significant profibrotic cytokine because it stimulates ECM synthesis and inhibits degradation^9^. Previous studies have reported that inhibition of the TGF-β1 signaling pathway attenuates fibrosis development^10^. Increasing evidence suggests that the functional Wnt/β-catenin pathway participates in HSCs activation and leads to liver fibrosis^11^. MAPK, another important cell signal involved in the proliferation and activation of HSCs, can lead to an aggravation of hepatic fibrosis and regulate many cellular functions, including apoptosis, cell proliferation, oxidative stress and inflammation^12^.

The augmentation of HSCs apoptosis promotes the resolution of fibrosis^13^. Cellular senescence, which is a stable form of cell cycle arrest, may limit the proliferative potential of premalignant cells. Recent studies have demonstrated that senescent cells exhibit large and flattened morphology, and accumulate senescence- associated β-galactosidase (SA-β-gal) activity. In addition, senescent cells activate the p53 and p16/Rb tumor suppressor pathways to promote senescence, which is consistent with the role of cellular senescence as an obstacle for transformation^14^. Senescent activated HSCs can down-regulate ECM production to limit liver fibrosis^15^.

The underlying mechanisms of liver fibrosis have been studied extensively, but there are no ideal drugs to treat this disease^16^. Therefore, it is necessary to develop new and efficient drugs for the treatment of liver fibrosis. Traditional Chinese medicines (TCMs) and natural products are abundant sources of biologically active substances for the treatment of human diseases^17^,^18^. Some active compounds, including astragaloside IV, uroslic acid and genistein, are highly effective against liver fibrosis^19^,^20^,^21^. Therefore, it is reasonable to develop effective candidates from medicinal plants for the treatment of liver fibrosis.

Dioscin (Dio, shown in Supplemental Figure 1) is a plant steroid saponin that is widely prevalent in many herbs^22^,^23^. Pharmacological investigations have demonstrated that dioscin possesses anti-inflammatory, lipid-lowering and anti-cancer activities^24^,^25^,^26^. Our previous works revealed that dioscin has potent effects against alcohol-induced liver injury and nonalcoholic fatty liver disease^27^,^28^. However, the effect of this compound against hepatic fibrosis has not been reported to the best of our knowledge.

Therefore, the present paper investigated the effects of dioscin against hepatic fibrosis and elucidated the potential underlying molecular mechanisms.

## Results

### Identification of primary HSCs

The purity of primary rat HSCs was confirmed using vitamin A autofluorescence and immunofluorescent staining of desmin. The level of α-SMA was significantly increased in primary HSCs cultured for 10 days compared with the cells cultured for 1 day, and the purities of the activated HSCs isolated from healthy and CCl_4_-treated rats were over 95% (Supplemental Figure 2A–D).

### Dioscin inhibits HSCs viability, but not hepatocyte

**Figure 1:**
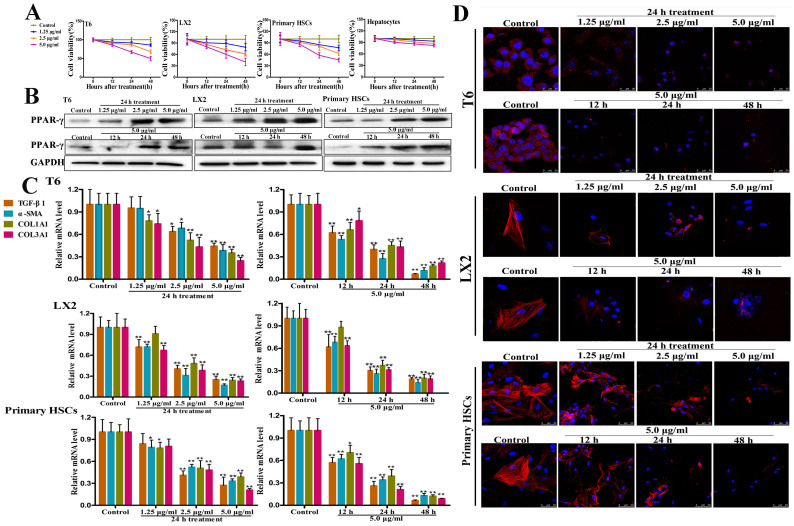
Effects of dioscin on HSCs activation of HSC-T6, LX2 and primary HSCs. (A) Impacts of dioscin on the cell viabilities of HSC-T6, LX2, primary rat HSCs. After incubating for 24 14h, the cells were treated with various concentrations of dioscin (1.25, 2.5 and 5.0 14μg/ml) or 0.1% DMSO as a negative control for 12, 24 and 48 14h, then the cell viability was evaluated by MTT assay. (B) Effects of dioscin on PPAR-γ protein expression in HSC-T6, LX2 and primary rat HSCs. The cells were treated with various concentrations of dioscin for 12, 24 and 48 14h. Total protein samples extracted from control and dioscin-treated HSCs were analyzed by western blotting assay (The cropped gels are used and full-length gels are presented in Supplemental Figure S9). (C) Effects of dioscin on the mRNA levels of TGF-β1, α-SMA, COL1A1 and COL3A1 in HSC-T6, LX2 and primary rat HSCs. The cells were treated with various concentrations of dioscin for 12, 24 and 48 14h, and then total RNA samples were extracted from control and dioscin-treated HSCs, which were measured by real-time PCR assay. (D) Effects of dioscin on the levels of α-SMA in HSC-T6, LX2 and primary rat HSCs based on immunofluorescence assay (10000 × magnification). After treatment with various concentrations of dioscin for 12, 24 and 48 14h, the expression of α-SMA was detected by immunofluorescence, and DAPI was used to visualize the nucleus. Data are presented as the mean ± SD (n ≥ 3). *p < 0.05 and **p < 0.01 compared with the control group.

Figure 1A demonstrates that dioscin significantly inhibited the viability of HSC-T6, LX2 and primary activated HSCs with dose- and time-dependent manners. Dioscin at the concentration of 5 14μg/ml for 48 14h significantly decreased the cell viabilities to 49.84%, 40.52% and 45.26%, respectively. Notably, dioscin at concentrations of 1.25, 2.5 and 5.0 14μg/ml for 12, 24 and 48 14h did not obviously reduce the viability of primary cultured hepatocytes, suggesting that the inhibition of cell viability induced dioscin was specific to HSCs.

### Dioscin inhibits HSCs activation in culture

Figure 1B demonstrates that dioscin markedly increased the expression of PPAR-γ, one marker of quiescent HSCs. In addition, the mRNA levels of some genes associated with HSCs activation were also assayed in HSC-T6, LX2 and primary HSCs. As shown in Figure 1C, dioscin greatly reduced TGF-β1, α-SMA, COL1A1 and COL3A1 mRNA levels, and also significantly reduced α-SMA level (Figure 1D). Furthermore, we also observed that the primary rat HSCs treated with 1.25 14μg/ml of dioscin at day 10 showed a more quiescent phenotype accompanied by the reduction of α-SMA compared with control HSCs cultures (Supplemental Figure 2E). Then, we removed dioscin after 10 days to investigate whether the HSCs could transdifferentiate into myofibroblasts again. Three days after dioscin removal, high expression of α-SMA was found in the cells with the typical myofibroblastic morphology, suggesting that dioscin inhibited HSCs activation in the present work.

### Dioscin induces HSCs apoptosis

**Figure 2:**
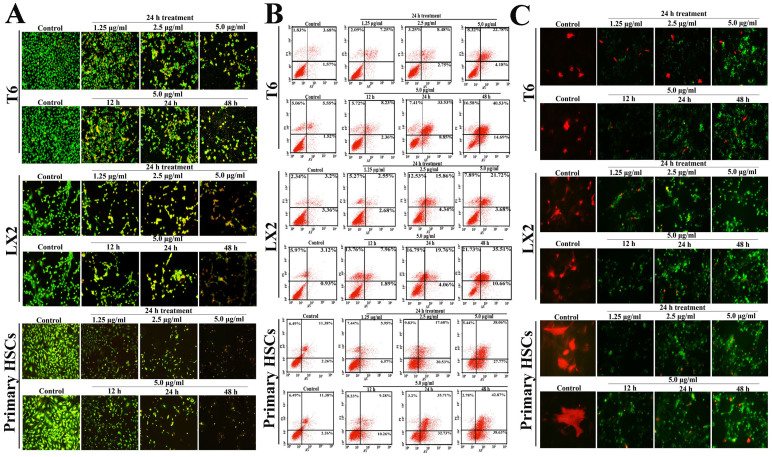
Dioscin induced apoptosis of HSC-T6, LX2 and primary HSCs. (A) Effects of dioscin on apoptosis of HSC-T6, LX2 and primary HSCs based on AO/EB staining, which were treated with 5.0 14μg/ml of dioscin for 12, 24 and 48 14h, or treated with different concentrations of dioscin (1.25, 2.5 and 5.0 14μg/ml) for 24 14h (200×, final magnification). (B) After being treated with 5.0 14μg/ml of dioscin for 12, 24 and 48 14h, or treated with different concentrations of dioscin (1.25, 2.5 and 5.0 14μg/ml) for 24 14h, HSC-T6, LX2 and primary HSCs were stained with annexin V/PI, and then analyzed by flow cytometry for quantitative detection of cell apoptosis. (C) HSC-T6, LX2 and primary HSCs were incubated with 5.0 14μg/ml of dioscin for 12, 24 and 48 14h, or treated with different concentrations of dioscin (1.25, 2.5 and 5.0 14μg/ml) for 24 14h, then the cells were dual-stained for TUNEL (green) and α-SMA (red) (original magnification 200×).

Dioscin caused the activated HSCs to undergo apoptosis based on AO/EB fluorescent staining (Figure 2A) and flow cytometry assay (Figure 2B). In the dual-staining for α-SMA and TUNEL, α-SMA-positive cells were obviously decreased and the numbers of TUNEL-positive cells were significantly increased by dioscin (Figure 2C). In contrast to the activated HSCs, dioscin hardly induced the apoptosis in the quiescent HSCs following incubation with 5.0 14μg/mL of diosicn for 24 14h. These results clearly indicated that dioscin induced apoptosis in activated, but not in quiescent HSCs *in vitro* (Supplemental Figure 2F). Dioscin- treated HSC-T6, LX2 and primary HSCs exhibited cell shrinkage and a loss of the originally confluent monolayer (Supplemental Figure 3). In addition, single cell gel electrophoresis evaluated the extent of dioscin-induced cellular DNA damage. Supplemental Figure 4A demonstrates that nuclei were intact and round and that the DNA remained within the nuclear matrix in control cells. However, obvious DNA damage was observed in dioscin-treated cells. Furthermore, HSCs cells treated with dioscin (5.0 14μg/ml) exhibited fewer cell surface microvilli, chromatin condensation, shrinkage of the cell nuclei and dilated endoplasmic reticulums in apoptotic cells on TEM assays (Supplemental Figure 4B).

### Inhibitory effect of dioscin on CCl_4_-induced hepatotoxicity

**Figure 3:**
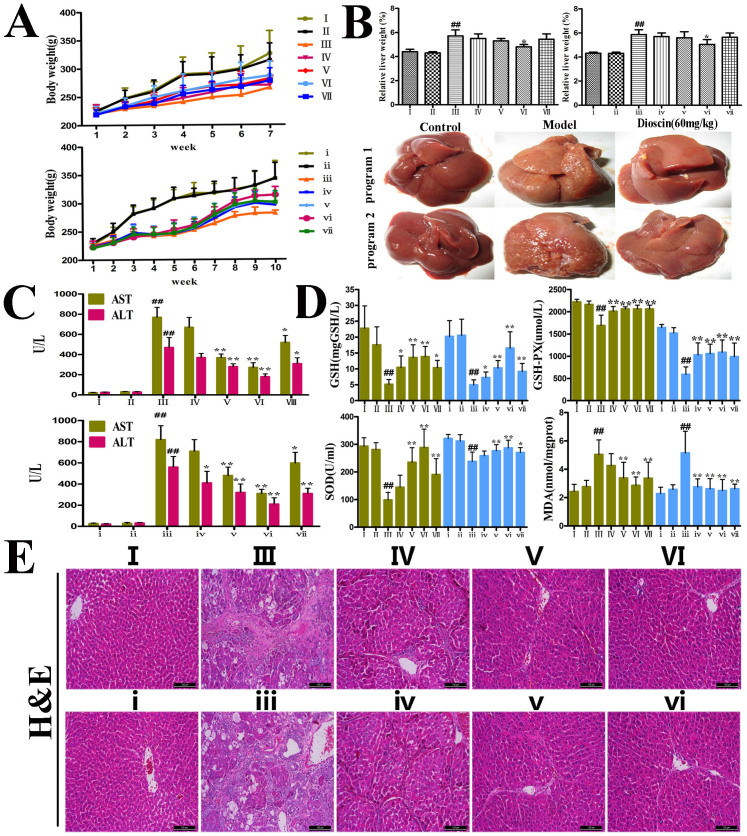
Inhibitory effects of dioscin on CCl_4_-induced hepatotoxicity. In program I, the rats were injected twice a week with CCl_4_ during 7 weeks in the presence or absence of dioscin, and in program II, CCl_4_ was injected for 10 weeks without dioscin, or after 4 weeks of CCl_4_, dioscin was administered together with CCl_4_ for additional times. (A–B) Effects of dioscin on rat body weights and livers. (C) Effects of dioscin on serum AST and ALT activities in rats as a measure for liver injury. (D) Effects of dioscin on GSH, GSH-Px, SOD and MDA levels. (E) H&E staining of representative liver sections (magnification, 100×). Values are expressed as the mean ± SD (n ≥ 3). *p < 0.05, **p < 0.01 vs. model group; ^##^p < 0.01 vs. normal control group.

Hepatic fibrosis in rats was produced by intraperitoneal (i.p.) injection of CCl_4_ for 7 or 10 weeks. Dioscin mixed with 0.5% carboxymethylcellulose (CMC-Na) was administered intragastrica- lly (i.g.) at the doses of 20, 40 and 60 14mg/kg once daily. Our *in vivo* tests showed that the body weights of rats in model groups were lower than those of in control groups (Figure 3A). However, dioscin significantly prevented the decrease in body weights relative to model groups. As shown in Figure 3B, the livers from CCl_4_-treated rats were puffy and stiff, and acquired an irregular and granular surface, which were all ameliorated by dioscin. In addition, the relative liver weight (%) was also reduced in dioscin-treated rats. The serum AST and ALT activities in CCl_4_-treated rats were markedly increased, which were also significantly suppressed by the compound (Figure 3C).

### Dioscin inhibits CCl_4_-induced oxidative liver injury

Figure 3D and Supplemental Figure 5 demonstrate that GSH, GSH-Px and SOD levels in the dioscin-treated groups (60 14mg/kg) increased significantly relative to the CCl_4_-treated groups. However, the levels of MDA, NO and iNOS in the dioscin-treated groups (60 14mg/kg) decreased markedly compared with the model groups.

### Dioscin attenuates liver fibrosis *in vivo*

**Figure 4:**
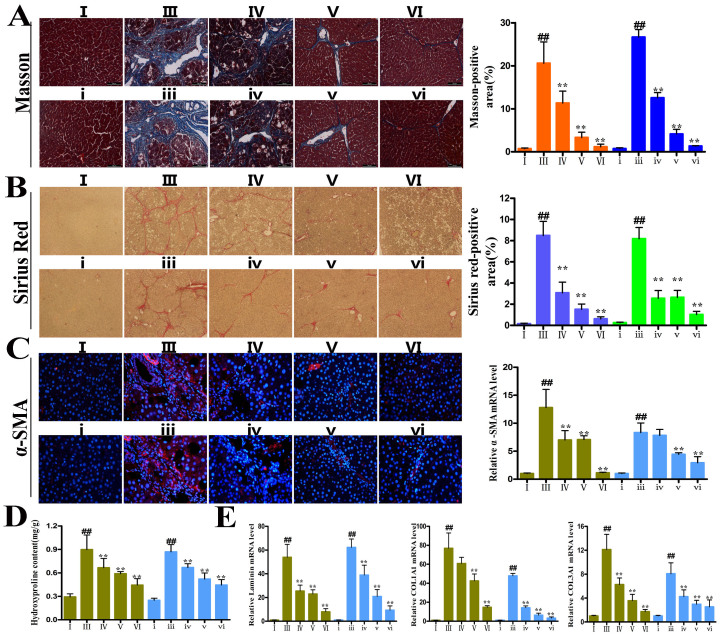
Dioscin attenuated liver fibrosis *in vivo*. (A–B) Effects of dioscin on liver fibrosis by Masson and Sirius Red staining (magnification 100×). (C) Effects of dioscin on expression of α-SMA by immunostaining in liver tissues extracted from rats treated with CCl_4_ and dioscin for 7 weeks and 10 weeks (magnification 200×) (D) Effects of dioscin on hydroxyproline levels in rat livers. (E) Effects of dioscin on the mRNA levels of laminin, COL1A1 and COL3A1 in rats. The results are expressed as mean ± SD (n ≥ 3). *p < 0.05, **p < 0.01 vs. model group; ^##^p < 0.01 vs. normal control group.

The livers from the control groups exhibited a normal lobular architecture, whereas samples from CCl_4_-treated groups exhibited severe hemorrhagic necrosis and inflammatory cell infiltration, which was dramatically attenuated by dioscin (Figure 3E). Dioscin also significantly decreased collagen fiber deposition based on Masson and Sirius Red staining (Figure 4A–B). Figure 4C demonstrates that the expression of α-SMA in dioscin-treated rats decreased significantly relative to CCl_4_-treated rats. These results were further confirmed by the levels of hepatic hydroxyproline (Figure 4D), laminin, Col1A1 and Col3A3 (Figure 4E) and by TEM assay (Supplemental Figure 6).

### Dioscin inhibits ECM accumulation by altering the levels of MMPs and TIMPs

**Figure 5:**
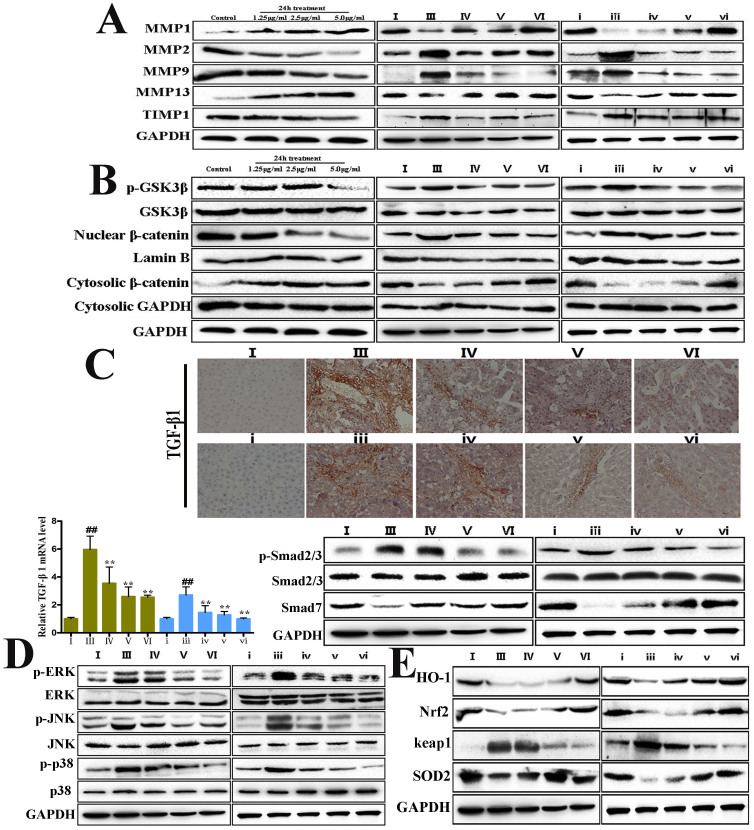
Dioscin ameliorated liver fibrosis by regulating MMPs/TIMPs, and altering Wnt/β-catenin, TGF-β/Smad and MAPK pathways and oxidative stress. (A) Effects of dioscin on the levels of MMP-1, MMP-2, MMP-9, MMP-13 and TIMP-1 from primay rat HSCs and *in vivo* HSCs isolated from normal, model and dioscin-treated rats. (B) Effects of dioscin on the levels of p-GSK3β, GSK3β, nuclear and cytosolic β-catenin in primay rat HSCs, and *in vivo* HSCs isolated from normal, model and dioscin-treated rats. (C) Effects of diocsin on expression of TGF-β1 by immunohistochemistry in liver tissue (magnification, 100×), and the levels of p-Smad2/3, Smad2/3 and Smad7 *in vivo* HSCs isolated from normal, model and dioscin-treated rats. (D) Effects of dioscin on the levels of MAPK phosphorylation *in vivo* HSCs isolated from normal, model and dioscin-treated rats. (E) Effects of dioscin on the levels of HO-1, Nrf2, keap1 and SOD2 *in vivo* HSCs isolated from normal, model and dioscin-treated rats. The cropped gels are used and full-length gels are presented in Supplemental Figure S10, S11, S12, S13 and S14. Values are expressed as the means ± SD (n = 3). *p < 0.05, **p < 0.01 vs. model group; ^##^p < 0.01 vs. normal control group.

As shown in Figure 5A, dioscin significantly decreased the levels of MMP-2, MMP-9, TIMP-1, and increased the levels of MMP-1 and MMP-13 in primary rat HSCs and *in vivo* the isolated HSCs from dioscin-treated rats compared with CCl_4_-treated animals (the results of statistical analysis are provided in Supplemental Figure 7).

### Dioscin inhibits the Wnt/β-catenin signaling pathway

As shown in Figure 5B, dioscin administration significantly down-regulated the levels of nuclear β-catenin and up-regulated the levels of cytosolic β-catenin in primary rat HSCs and *in vivo* the isolated HSCs from dioscin-treated rats compared with CCl_4_-treated animals. However, total GSK3β protein levels were not altered, and dioscin markedly reduced phospho- GSK3β protein levels (the results of statistical analysis are provided in Supplemental Figure 7).

### Dioscin affects the TGF-β/smad signaling pathway

As shown in Figure 5C, immunohistochemical staining and real-time PCR assay revealed the elevated TGF-β1 expression in the isolated activated HSCs from CCl_4_-treated rats, which was significantly suppressed by dioscin. The levels of p-Smad2 were obviously reduced. In addition, dioscin up-regulated Smad7 expression relative to the activated HSCs isolated from CCl_4_-treated rats.

### Dioscin regulates the MAPK signaling pathway

As shown in Figure 5D, the activated HSCs isolated from CCl_4_-treated rats significantly increased the levels of phosphorylated ERK, JNK and p38, which were restored by dioscin.

### Dioscin attenuates CCl_4_-induced oxidative stress and inflammation

**Figure 6:**
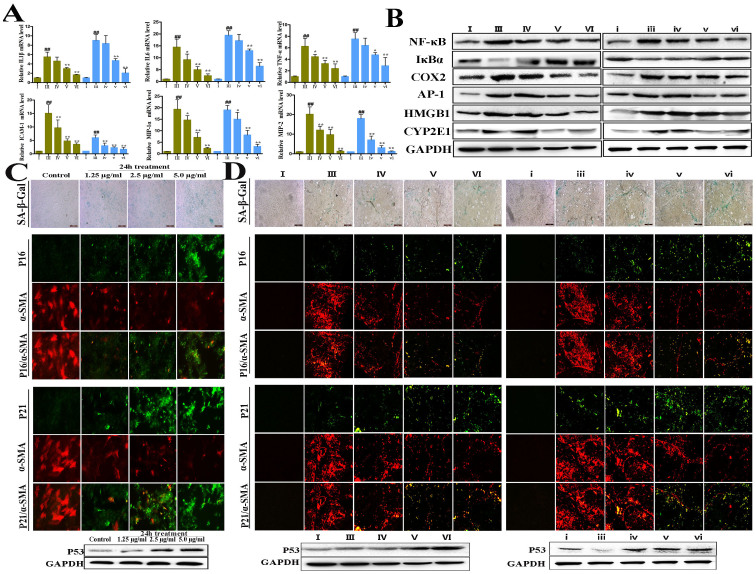
Dioscin reduced inflammation and induced senescence of activated HSCs. (A) Effects of dioscin on the mRNA levels of IL-1β, IL-6, TNF-α, ICAM-1, MIP-1α and MIP-2 *in vivo* HSCs isolated from normal, model and dioscin-treated rats. (B) Effects of dioscin on expression levels of NF-κB, IκBα, COX2, AP-1, HMGB1 and CYP2E1 *in vivo* HSCs isolated from normal, model and dioscin-treated rats. (C) Effects of dioscin on cell senescence in primary HSCs treated with different concentrations of dioscin (24 14h) for SA-β-Gal staining and dual-staining for p16 or p21 (green) and α-SMA (red) assay (original magnification 200×). (D) Effects of dioscin on cell senescence in live tissues from the rats treated with CCl_4_ and dioscin for SA-β-Gal staining and double immunostaining of p16/α-SMA and p21/α-SMA (original magnification 200×). The cropped gels are used and full-length gels are presented in Supplemental Figure S15 and S16. Values are expressed as the mean ± SD (n = 3). *p < 0.05, **p < 0.01 vs. model group; ^##^p < 0.01 vs. normal control group.

The levels HO-1, Nrf2 and SOD2 were significantly decreased in the isolated activated HSCs from CCl_4_-treated rats, whereas keap1 level was markedly increased (Figure 5E). However, dioscin treatment significantly elevated HO-1, Nrf2 and SOD2 levels and decreased keap1 level. Additionally, the levels of IL-1β, IL-6, TNF-α, ICAM-1, MIP-1α and MIP-2 in model groups were significantly increased compared with the HSCs isolated from control animals, and these changes were reversed by dioscin (Figure 6A). Dioscin also markedly reduced the levels of NF-κB, COX2, AP-1, HMGB1 and CYP2E1 and increased IκBα level compared with model groups (Figure 6B).

### Dioscin induces activated HSCs senescence

Figure 6C-D demonstrates that the primary rat HSCs and the livers from dioscin-treated rats exhibited significantly higher numbers of SA-β-gal-positive cells. Dioscin also elevated the numbers of p16- and p21-positive cells and reduced α-SMA-positive cells *in vitro* and *in vivo*. In addition, dioscin significantly increased p53 expression.

### Dioscin induces apoptosis of primary HSCs *in vivo*

**Figure 7:**
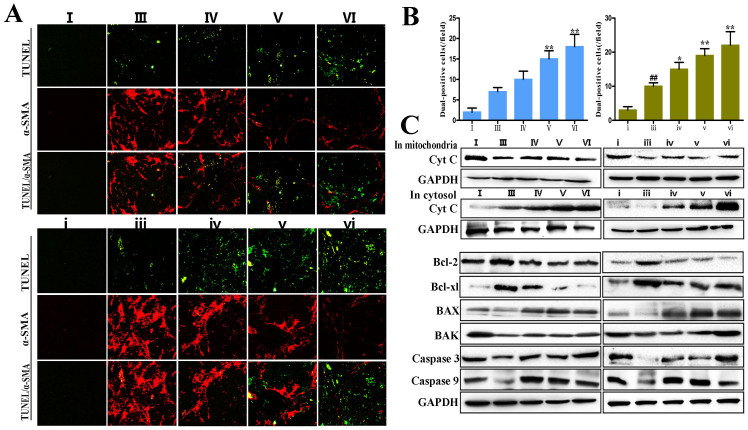
Dioscin induced apoptosis of activated HSCs *in vivo*. (A) Effects of dioscin on liver tissues from the rats treated with CCl_4_ and dioscin dual-stained for TUNEL (green) and α-SMA (red) assay (original magnification 200×). (B) Numbers of α-SMA and TUNEL dual-positive cells was counted in five random fields, and the average numbers of dual-positive cells were plotted. (C) Effects of dioscin on cytochrome c release, and the levels of Bcl-2, Bcl-xl, BAX, BAK, caspase 3 and caspase 9 *in vivo* HSCs isolated from normal, model and dioscin-treated rats. The cropped gels are used and full-length gels are presented in Supplemental Figure S17. Values are expressed as the mean ± SD (n = 3). *p < 0.05, **p < 0.01 vs. model group; ^##^p < 0.01 vs. normal control group.

Figure 7A demonstrates that the numbers of TUNEL-positive cells were significantly elevated and the α-SMA-positive cells were obviously decreased in the livers of dioscin-treated rats. In fact, the number of dual-positive cells per field in dioscin-treated rats was nearly 2-fold higher than that of in CCl_4_-treated rats (Figure 7B), which clearly indicated that dioscin induced apoptotic cell death in activated HSCs *in vivo*. Furthermore, the release of cytochrome c from mitochondria to the cytoplasm was obvious in dioscin- treated groups. In addition, freshly isolated activated HSCs from CCl_4_-treated rats exhibited an up-regulation of Bcl-2 and Bcl-xl and a down-regulation of BAX, BAK, caspase-3 and caspase-9 relative to cells isolated from control animals, which were significantly restored by dioscin (Figure 7C).

## Discussion

Liver fibrosis, a precursor of cirrhosis, results from chronic liver injury. Dioscin, one active natural product, shows the hepatoprotective effects against CCl_4_-induced acute liver injury in our works^24^,^25^,^26^,^27^,^28^,^29^. In the present paper, dioscin exhibited excellent effects against CCl_4_-induced chronic liver damage through decreasing the levels of AST and ALT, preventing body weight reduction and ameliorating the distortion of normal architecture, extensive hemorrhagic necrosis and collagen deposition. These results suggest that dioscin attenuated liver fibrosis.

HSCs play an important role in the development of liver fibrosis^1^. Therefore, activated HSCs appear to be a specific cellular target for the treatment of liver fibrosis^30^. Here, the effects of dioscin against hepatic fibrosis were evaluated *in vitro* and *in vivo*, and the data showed that dioscin effectively inhibited the myofibroblast -like activation of HSCs, but no effect to the primary cultured rat hepatocytes. Recently, PPAR-γ has been recognized as a potential molecular target to inhibit HSC activation^31^. Interestingly, PPAR-γ was significantly up-regulated by dioscin in our work, and the levels of some typical HSCs activation markers, including α-SMA, laminin, COL1A1 and COL3A1 were all decreased by dioscin. These results suggest that dioscin inhibited HSCs activation.

ECM accumulation is a common phenomenon in liver fibrosis. Hydroxyproline, a major constituent of collagen, is a good marker of ECM accumulation^32^. Dioscin markedly decreased hydroxyproline content in the present work, which indicates that this compound inhibited ECM accumulation and suppressed hepatic fibrosis. MMPs, and their tissue inhibitors, TIMPs, are a class of secreted enzymes with important functions in ECM degradation^33^. Dioscin markedly down-regulated the levels of MMP-2, MMP-9, TIMP-1, and up-regulated the levels of MMP-1 and MMP-13. Thus, dioscin enhanced ECM degradation by regulating the balance of MMPs and TIMPs.

The induction of apoptosis in HSCs is a crucial mechanism to decrease the number of activated HSCs during the development of hepatic fibrosis^34^. Our *in vitro* study revealed that dioscin caused obvious morphological changes in activated HSC- T6, LX2 and primary HSCs, including cell shrinkage, nuclear condensation, membrane blebbing and fragmentation. Dioscin also increased the number of TUNEL -positive activated HSCs *in vivo*. The mitochondrial signaling pathway represents a central checkpoint for apoptosis, which can be regulated by members of the Bcl-2 family^35^,^36^. Recent studies have demonstrated that the over-expression of Bcl-2 in activated HSCs enhances the resistance to apoptosis, which may result in the development of fibrosis^37^. Cytochrome c is released from mitochondria to the cytosol after liver injury, which is essential for apoptosis induction and the activation of the caspase cascade^38^. Dioscin significantly increased cytochrome c release, decreased Bcl-2 and Bcl-xl expression and increased the levels of caspase-3, caspase-9, BAX and BAK in our study. These findings demonstrate that the effects of dioscin against liver fibrosis may be mediated through the induction of apoptosis in activated HSCs *in vitro* and *in vivo*.

Senescence of activated HSCs down-regulates ECM expression, which indicates that the senescence of activated HSCs limits liver fibrosis^15^. Dioscin significantly increased the number of SA-β-gal-positive cells, the number of positive cells that expressed senescence markers p16 and p21, and increased the levels of the senescence-associated marker p53. Therefore, the effects of dioscin against liver fibrosis may be associated with the increased senescence of activated HSCs.

Previous studies have demonstrated that inhibition of the TGF-β1 signaling pathway attenuates liver fibrosis^10^. Signaling of TGF-β family members is mediated by TGFβR, which phosphorylates downstream receptor-activated Smads^34^. Dioscin inhibited TGF-β1 expression, reduced TGF-β1-induced phosphorylation of Smad2, and increased Smad 7 expression in our study. These results indicate that that anti- fibrotic effect of dioscin may be mediated through the inhibition of the TGF-β1/Smad pathway.

Increasing evidence indicates that the Wnt/β-catenin pathway participates in HSCs activation, which leads to liver fibrosis^11^. Thus, we focused on the role of the Wnt/β- catenin pathway in CCl_4_-induced liver fibrosis. Dioscin significantly inhibited β-catenin nuclear translocation. However, dioscin did not alter total GSK3β protein levels but markedly decreased phospho-GSK3β. Recent evidence also suggests that canonical Wnt signaling exerts an anti-apoptotic effect in HSCs^39^. Therefore, we speculate that dioscin induced cell death of activated HSCs through inhibiting Wnt/β- catenin signaling.

Oxidative stress and consequent lipid peroxidation are involved in the generation of liver fibrosis^40^. Therefore, some oxidative stress parameters, including GSH, GSH-Px, SOD, MDA, NO and iNOS, were examined in this study. Dioscin significantly increased GSH, GSH-Px and SOD levels and markedly decreased MDA, NO and iNOS levels, which indicated that dioscin inhibited lipid peroxidation in liver fibrosis. In addition, CCl_4_-treated rats exhibited the changes in the balance between anti- oxidant and pro-oxidant activity, including increased keap1 expression and reduced Nrf2, HO-1 and SOD2 expression. Dioscin attenuated CCl_4_-induced hepatic oxidative stress in our work through the up-regulation of Nrf2, HO-1 and SOD2 and the down- regulation of keap1. Therefore, the anti-oxidant effects of dioscin might be attributable to its anti- fibrosis effect.

Chronic inflammatory reactions play an important role in the triggering of liver fibrosis^8^. Following inflammatory cell infiltration, pro-inflammatory cytokine over -production and increased oxidative stress induce hepatocyte damage directly. NF-κB and its associated inflammatory cascade play critically vital roles in hepatocyte survival and damage, HSCs and inflammatory cell activation, pro-inflammatory cytokine production, liver injury and fibrosis^41^,^42^. Our results demonstrate that dioscin suppressed pro-inflammatory cytokine production and NF-κB activation, including decreased hepatic NF-κB activity and increased IκBα, and down-regulated inflammatory cytokines including TNF-α, IL-1β, IL-6, ICAM-1, MIP-1α, MIP-2, COX-2, AP-1, HMGB1 and CYP2E1. These results indicate that the anti-fibrotic effect of dioscin may be mediated through inflammation suppression.

The MAPK family, including the three major subgroups (extracellular signal- regulated kinase (ERK), p38 MAPK (p38) and c-Jun N-terminal kinase/stress- activated protein kinase (JNK), is involved in the proliferation and activation of HSCs, and the aggravation of hepatic fibrosis^19^,^43^. MAPKs can regulate many cellular functions, including gene expression, immune response, cell apoptosis, proliferation, responses to oxidative stress and inflammation^12^. The results by Wang et al. have shown that blocking ERK1/2 can reverse Bcl-2 up-regulation and Bax down- regulation in activated HSCs^44^. In addition, a pathway of apoptosis induced by an ERK-dependent increased the activation of the downstream kinases JNK and p38 MAP kinase has been reported, and ERK-induced apoptosis occur with or without activation of JNK has also been investigated^45^,^46^. In the present work, dioscin down- regulated the levels of phosphorylated ERK, p38 and JNK, which is likely associated with the induction of cell death of activated HSCs.

In summary, the current study demonstrated that dioscin markedly attenuated liver fibrosis *in vitro* and *in vivo*. *In vitro*, dioscin inhibited HSCs activation and induced HSCs apoptosis. *In vivo*, dioscin decreased ECM accumulation, regulated the balance between collagen synthesis and degradation, induced apoptosis and senescence in activated HSCs, and prevented liver injury. The anti-fibrotic effect of dioscin may affect inflammation, oxidative stress, and TGF-β/smad, MAPK, mitochon -drial and Wnt/β-catenin signaling pathways. Our results suggest that dioscin is a potential agent for the treatment of liver fibrosis.

## Methods

### Tested drug

Dioscin with a purity >98% was prepared in our laboratory^47^,^48^. Dioscin was dissolved with 0.1% dimethylsulfoxide (DMSO) before use for all *in vitro* experiments or 0.5% CMC-Na solution for our *in vivo* tests. In addition, silymarin, a natural product with the potent effects against liver injury^49^,^50^, was purchased from Sigma Chemical Company (Milan, Italy) and used as the positive control in our *in vivo* experiments.

### Cell culture

Immortalized human (LX2) and rat (HSC-T6) cell lines with characteristics of an activated HSCs phenotype were used according to previous reports^51^,^52^. Primary HSCs were isolated from normal male Wistar rats (450–500 14g) using sequential pronase and collagenase perfusion as previously described^53^. We also isolated *in vivo*-activated HSCs using the same procedure from normal and fibrotic rats with or without dioscin. Hepatocytes were isolated from normal male Wistar rats (200 ± 20 14g) via *in situ* perfusion of livers using collagenase followed by differential centrifugations as described previously^54^. Different types of cells were cultured in DMEM supplemented with 10% FBS (GIBCO®, Invitrogen, Carlsbad, CA, USA), 100 14IU/ml penicillin and 100 14mg/ml streptomycin. Cultures were maintained at 37°C in a humidified air containing 5% CO_2_ for experiments.

### Cell viability assay

HSC-T6, LX2 and primary HSCs cells were seeded in a 96-well plate with 100 14μl (5 × 10^4^ 14cells/ml) per well and incubated for 24 14h before the addition of the stimulus. Cells were incubated for 12, 24 or 48 14h and treated with different concentrations of dioscin (1.25, 2.5 or 5.0 14μg/ml). The effects of dioscin on T6, LX2 and primary HSC viability were analyzed using an MTT assay. The cells treated with the same volume of 0.1% DMSO alone were used as the control.

### Immunofluorescence assay of α-SMA

HSC-T6, LX2, primary HSCs cells were plated in six-well plates overnight and treated as described above. The cells were fixed with 2% paraformaldehyde for 15 14min and incubated with 0.5% Triton X-100 for 15 14min. Cells were blocked with 4% BSA at room temperature for 2 14h and incubated with an anti-α-SMA antibody (1:100) at 4°C overnight. Cells were washed twice in PBS and incubated with a fluorescein- labeled secondary antibody for 1 14h. Cell nuclei were stained with DAPI (5 14μg/ml). Images of the cells were photographed using a laser scanning confocal microscope (Leica, TCS SP5, Germany).

### Apoptosis assay of HSCs

Acridine orange (AO) and ethidium bromide (EB) staining was used to distinguish live cells from dead ones in our work, and the living cells have green nuclei, while early apoptotic cells have yellow staining and irregular nuclei. In late apoptotic cells, EB penetrates the fractured cell membrane and stains the nucleus orange^55^. The HSC- T6, LX2 and primary HSCs cells were plated in six-well plates overnight and treated as described above. Cold PBS was used to remove any residual solvent after incubation. The cells were stained with AO/EB, and the cells were observed under a fluorescence microscope (OLYMPUS, Japan). In addition, the Annexin V-FITC Apoptosis Detection Kit (KeyGEN, Nanjing, China) was used to detect dioscin- induced HSCs apoptosis, and the samples were analyzed using flow cytometry (Becton-Dickinson, USA).

### α-SMA and TUNEL dual staining *in vitro*

HSC-T6, LX2 and primary HSCs cells were plated in twelve-well plates overnight and treated as described above. Then the cells were treated with an anti-α-SMA antibody (1:100) at 4°C overnight. After washing with PBS, the cells were incubated with a fluorescein-labeled secondary antibody for 1 14h at room temperature. After staining of α-SMA, the cells were then incubated with TUNEL reaction mixture at 37°C for 1 14h according to the manufacturer's instructions. Images were photographed using fluorescence microscopy (OLYMPUS, Japan).

### CCl_4_-induced rat hepatic fibrosis models

Male Wistar rats weighing 150–200 14g were provided by the Experimental Animal Center of Dalian Medical University, Dalian, China (Quality certificate number: SCXK (Liao) 2008–0002). All experimental procedures were approved by the Animal Care and Use Committee of Dalian Medical University and performed in strict accordance with the People's Republic of China Legislation Regarding the Use and Care of Laboratory Animals.

Program 1: The animals were randomly distributed into seven groups (n = 10): Group I (normal control group), in which the rats were orally administered vehicle only; Group II (dioscin control group), in which the rats were orally administered dioscin (60 14mg/kg); Group III (model group), in which the rats received an intraperi-toneal injection of CCl_4_ (1 14ml/kg, 50% CCl_4_/olive oil) twice weekly for 7 weeks; Groups IV-VI (dioscin-treated groups), in which the rats were orally administered dioscin at doses of 20, 40 or 60 14mg/kg, respectively, once daily for 7 consecutive weeks and simultaneously treated intraperitoneally with CCl_4_ for 7 weeks; and Group VII (positive control group), in which the rats were administered silymarin at a dose of 100 14mg/kg and simultaneously received CCl_4_ injections for 7 weeks (Supplemental Figure 8).

Program 2: The rats were divided into seven groups: Group I (control group); Group ii (dioscin control group), in which rats were orally administered dioscin at 60 14mg/kg; Group iii (model group), in which the rats received intraperitoneal injections of CCl_4_ twice weekly for 10 weeks; Group iv-vi (dioscin-treated groups), in which the rats were injected with CCl_4_ for 4 weeks and then administered dioscin at doses of 20, 40 or 60 14mg/kg daily, respectively, together with CCl_4_ injections for another 6 weeks; and Group vii (positive control group), in which rats were administered silymarin (100 14mg/kg) and were treated the same as the dioscin-treated groups (Supplemental Figure 8).

The body weights of the rats were measured once per week during the experimental process. The animals were sacrificed at the end of the tests. Blood samples were obtained for biochemical analyses. Liver specimens were fixed in 10% buffered formalin and embedded in paraffin.

### Biochemical assays

The levels of ALT, AST, GSH, GSH-PX, SOD, MDA, NO and iNOS were detected using detection kits (Jiancheng Institute of Biotechnology, Nanjing, China) based on the manufacturer's' instructions.

### Histological assays

Liver samples were fixed in formalin, paraffin-embedded and sectioned. Liver sections were stained with hematoxylin-eosin (H&E) for routine histological examination. Sections were stained with Masson's trichrome and Sirius Red stains to estimate liver fibrosis. The degree of liver fibrosis was quantified using Image-Pro Plus 6.0 software.

### Hydroxyproline determination

Hydroxyproline levels in rat livers were measured using a detection kit (Jiancheng Institute of Biotechnology, Nanjing, China) based on the manufacturer's instructions. The results are reported as milligrams of hydroxyproline per gram of wet liver tissue.

### Immunohistochemical staining of TGF-β1

Paraffin-fixed liver tissue slices were sectioned, deparaffinized, rehydrated, and immersed in 3% H_2_O_2_ for 10 14min to block endogenous peroxidase activity. Antigen retrieval was performed in citrate buffer (pH = 6.0) in a microwave oven for 15 14min. BSA (5%) was used to block non-specific protein binding. The sections were incubated with a TGF-β1 primary antibody overnight at 4°C. The sections were washed with PBS, incubated with a biotinylated secondary antibody followed by an avidin-biotin-peroxidase complex, and stained with DAB. Images were acquired using light microscopy (Nikon Eclipse TE2000-U, NIKON, Japan).

### Senescence-associated β-galactosidase (SA-β-Gal) assay

Paraffin-fixed liver specimens were deparaffinized and rehydrated. The sections were stained using a senescence-associated β-galactosidase staining kit (Beyotime Biotechn -ology, China).

### P16/α-SMA and P21/α-SMA dual staining

Dual staining was carried out using primary antibodies against p16 and α-SMA, or p21 and α-SMA in primary rat HSCs and liver tissue slices of normal, model and dioscin-treated groups at 4°C overnight. Then the cells and liver tissue slices were incubated at 37°C for 1 14h with the corresponding secondary antibodies. Images were photographed using fluorescence microscopy (OLYMPUS, Japan).

### α-SMA and TUNEL dual staining *in vivo*

The liver tissue slices of normal, model and dioscin-treated groups were incubated with primary antibody against α-SMA. After washing with PBS, the sections were incubated with a fluorescein-labeled secondary antibody. After staining α-SMA, the sections were then incubated with TUNEL reaction mixture. Images were photogra -phed using fluorescence microscopy (OLYMPUS, Japan).

### Real-time PCR assay

Total RNA samples from HSC-T6, LX-2 and primary rat HSCs, and *in vivo* HSCs isolated from normal, model and dioscin-treated rats were extracted using RNAiso Plus reagent following the manufacturer's protocols. RNA (1 14μg) was reverse- transcribed using a PrimeScript® RT reagent Kit in a TC-512 PCR system (TECHNE, UK), and single-stranded cDNA was quantified using real-time PCR with SYBR® PremixEx Taq™II (Tli RNaseH Plus) in an ABI 7500 Real-Time PCR System (Applied Biosystems, USA). The primers used in the present work are listed in Supplemental Table 1. A no-template control was analyzed in parallel with each gene, and the GAPDH gene was used as the house-keeping gene in our study. The unknown template was calculated using a standard curve for quantitative analysis.

### Western blotting assay

Total protein samples were extracted from HSC-T6, LX-2 and primary rat HSCs, and *in vivo* HSCs isolated from normal, model and dioscin-treated rats using an appropriate cold lysis buffer supplemented with 1 14mM phenylmethylsulfonyl fluoride (PMSF), and the protein concentration was determined using a BCA protein assay kit (Beyotime Biotechnology, China). Samples were subjected to SDS-PAGE (10%–15%) and transferred onto a PVDF membrane (Millipore, USA). Membranes were blocked and incubated overnight at 4°C with the primary antibodies listed in Supplemental Table 2. Membranes were incubated at room temperature with an appropriate secondary antibody, and proteins were detected using an enhanced chemiluminesc -ence (ECL) method. Protein bands were imaged using a Bio-Spectrum Gel Imaging System (UVP, USA). Bands were normalized with GAPDH as an internal control.

### Statistical analysis

Data are reported as the mean ± SD. Comparisons were performed using one-way ANOVA. Statistical calculations were performed using SPSS Statistics 13.0 (IBM, New York, USA). Statistical significance was set at p < 0.05 or p < 0.01.

## Supplementary Material

Supplementary Information Supplementary Information
