# Supplementary material for: Potent effects of dioscin against liver fibrosis
Source: Sci Rep. 2015 Apr 8;5:9713. doi: 10.1038/srep09713 (PMC4389718; doi:10.1038/srep09713)
Supplement: Supplementary Information [file srep09713-s1.doc]

**Potent effects of dioscin against liver fibrosis**

Xiaoling Zhang, Xu Han, Lianhong Yin, Lina Xu, Yan Qi, Youwei Xu, Huijun Sun, Yuan Lin, Kexin Liu, Jinyong Peng*

*College of Pharmacy, Dalian Medical University, Western 9 Lvshunnan Road,*

*Dalian 116044, China*

Corresponding author: Dr. Jinyong Peng

Address correspondence: College of Pharmacy, Dalian Medical University, Western 9 Lvshunnan Road, Dalian 116044, China. Tel.: +86 411 8611 0411; Fax: +86 411 8611 0411; Email: jinyongpeng2008@126.com.

**Supplemental Table 1.** The primer sequences used for real-time PCR assay

| Gene | GenBank accession | Forward primer (5'-3') | reverse primer(5'-3') |
| --- | --- | --- | --- |
| Human TGF-β1 | NM_000660.4 | CGCATCCTAGACCCTTTCTCCTC | GGTGTCTCAGTATCCCACGGAAAT |
| Rat TGF-β1 | NM_021578 | CATTGCTGTCCCGTGCAGA | AGGTAACGCCAGGAATTGTTGCTA |
| Human α-SMA | NM_001141945.1 | ATAGAACATGGCATCATCACCAAC | GGGCAACACGAAGCTCATTGTA |
| Rat α-SMA | NM_031004 | AGCCAGTCGCCATCAGGAAC | GGGAGCATCATCACCAGCAA |
| Human COL1A1 | NM_000088 | CCATCAAAGTCTTCTGCAACATG | CGCCATACTCGAACTGGAATC |
| Rat COL1A1 | NM_053304 | GACATGTTCAGCTTTGTGGACCC | AGGGACCCTTAGGCCATTGTGTA |
| Human COL3A1 | NM_000090 | TGGATGGTGGTTTTCAGTTTAGCTA | TTTACATTTCCACTGGCCTGATC |
| Rat COL3A1 | NM_032085 | TTTGGCACAGCAGTCCAATGTA | GACAGATCCCGAGTCGCAGA |
| Rat laminin | NM_001108237 | GAATCAGCAGTGCCAAAGTGGA | CACCGTTGTTGACGTGAAATGAG |
| Rat IL1-β | NM_031512.2 | CCCTGAACTCAACTGTGAAATAGCA | CCCAAGTCAAGGGCTTGGAA |
| Rat IL-6 | NM_012589.1 | ATTGTATGAACAGCGATGATGCAC | CCAGGTAGAAACGGAACTCCAGA |
| Rat TNF-α | NM_012675.3 | TCAGTTCCATGGCCCAGAC | GTTGTCTTTGAGATCCATGCCATT |
| Rat ICAM-1 | [NM_012967.1](http://www.ncbi.nlm.nih.gov/sites/entrez?cmd=search&db=nucleotide&dopt=GenBank&term=NM_012967.1) | GCTTCTGCCACCATCACTGTGTA | ATGAGGTTCTTGCCCACCTG |
| Rat MIP-1α | NM_053647.1 | TCATGAAGTTTGTCTCAACCCTGAA | AGACAGCGAGGCACATCAGGTA |
| Rat MIP-2 | NM_013025.2 | GTTACCTGCTCAGCACCATGAA | GCAAAGGCTGCTGGTCTCAA |
| Rat GAPDH | NM_017008.3 | GGCACAGTCAAGGCTGAGAATG | ATGGTGGTGAAGACGCCAGTA |
| Human GAPDH | NM_002046.3 | GCACCGTCAAGGCTGAGAAC | TGGTGAAGACGCCAGTGGA |

**Supplemental Table 2.** The information of the antibodies used in the present work

| Antibody | Source | Dilutions | Company |
| --- | --- | --- | --- |
| α-SMA | rabbit | 1: 100 | Proteintech Group, Chicago, USA |
| P16 | rabbit | 1: 100 | Proteintech Group, Chicago, USA |
| P21 | rabbit | 1: 100 | Proteintech Group, Chicago, USA |
| PPAR-γ | rabbit | 1: 1000 | Proteintech Group, Chicago, USA |
| MMP-1 | rabbit | 1: 1000 | Proteintech Group, Chicago, USA |
| MMP-2 | rabbit | 1: 1000 | Proteintech Group, Chicago, USA |
| MMP-9 | rabbit | 1: 1000 | Proteintech Group, Chicago, USA |
| MMP-13 | rabbit | 1: 1000 | Proteintech Group, Chicago, USA |
| TIMP-1 | rabbit | 1: 1000 | Proteintech Group, Chicago, USA |
| p-GSK3β | rabbit | 1: 1000 | Proteintech Group, Chicago, USA |
| GSK3β | rabbit | 1: 1000 | Proteintech Group, Chicago, USA |
| β-catenin | rabbit | 1: 1000 | Proteintech Group, Chicago, USA |
| p-Smad2 | rabbit | 1: 1000 | Bioworld Technology, USA |
| Smad2 | rabbit | 1: 1000 | Proteintech Group, Chicago, USA |
| Smad7 | rabbit | 1: 1000 | Abcam, United States |
| p-ERK | rabbit | 1: 500 | Bioworld Technology, USA |
| ERK | rabbit | 1: 500 | Bioworld Technology, USA |
| p-JNK | rabbit | 1: 500 | Bioworld Technology, USA |
| JNK | rabbit | 1: 500 | Bioworld Technology, USA |
| p-p38 | rabbit | 1: 500 | Bioworld Technology, USA |
| p38 | rabbit | 1: 500 | Bioworld Technology, USA |
| HO-1 | rabbit | 1: 1000 | Bioworld Technology, USA |
| Nrf2 | rabbit | 1: 1000 | Bioworld Technology, USA |
| keap1 | rabbit | 1: 1000 | Proteintech Group, Chicago, USA |
| SOD2 | rabbit | 1: 1000 | Proteintech Group, Chicago, USA |
| NF-κB | rabbit | 1: 1000 | Proteintech Group, Chicago, USA |
| IκBα | rabbit | 1: 1000 | Proteintech Group, Chicago, USA |
| COX2 | rabbit | 1: 1000 | Proteintech Group, Chicago, USA |
| AP-1 | rabbit | 1: 1000 | Proteintech Group, Chicago, USA |
| HMGB1 | rabbit | 1: 1000 | Proteintech Group, Chicago, USA |
| CYP2E1 | rabbit | 1: 1000 | Proteintech Group, Chicago, USA |
| Cytochrome c | rat | 1: 1000 | Beyotime Biotechnology, China |
| Bcl-2 | rabbit | 1: 1000 | Proteintech Group, Chicago, USA |
| Bcl-xl | rabbit | 1: 1000 | Proteintech Group, Chicago, USA |
| BAX | rabbit | 1: 1000 | Proteintech Group, Chicago, USA |
| BAK | rabbit | 1: 1000 | Proteintech Group, Chicago, USA |
| Caspase 3 | rabbit | 1: 1000 | Proteintech Group, Chicago, USA |
| Caspase 9 | rabbit | 1: 1000 | Proteintech Group, Chicago, USA |
| GAPDH | rat | 1: 1000 | Proteintech Group, Chicago, USA |
| GAPDH | rabbit | 1: 1000 | Proteintech Group, Chicago, USA |


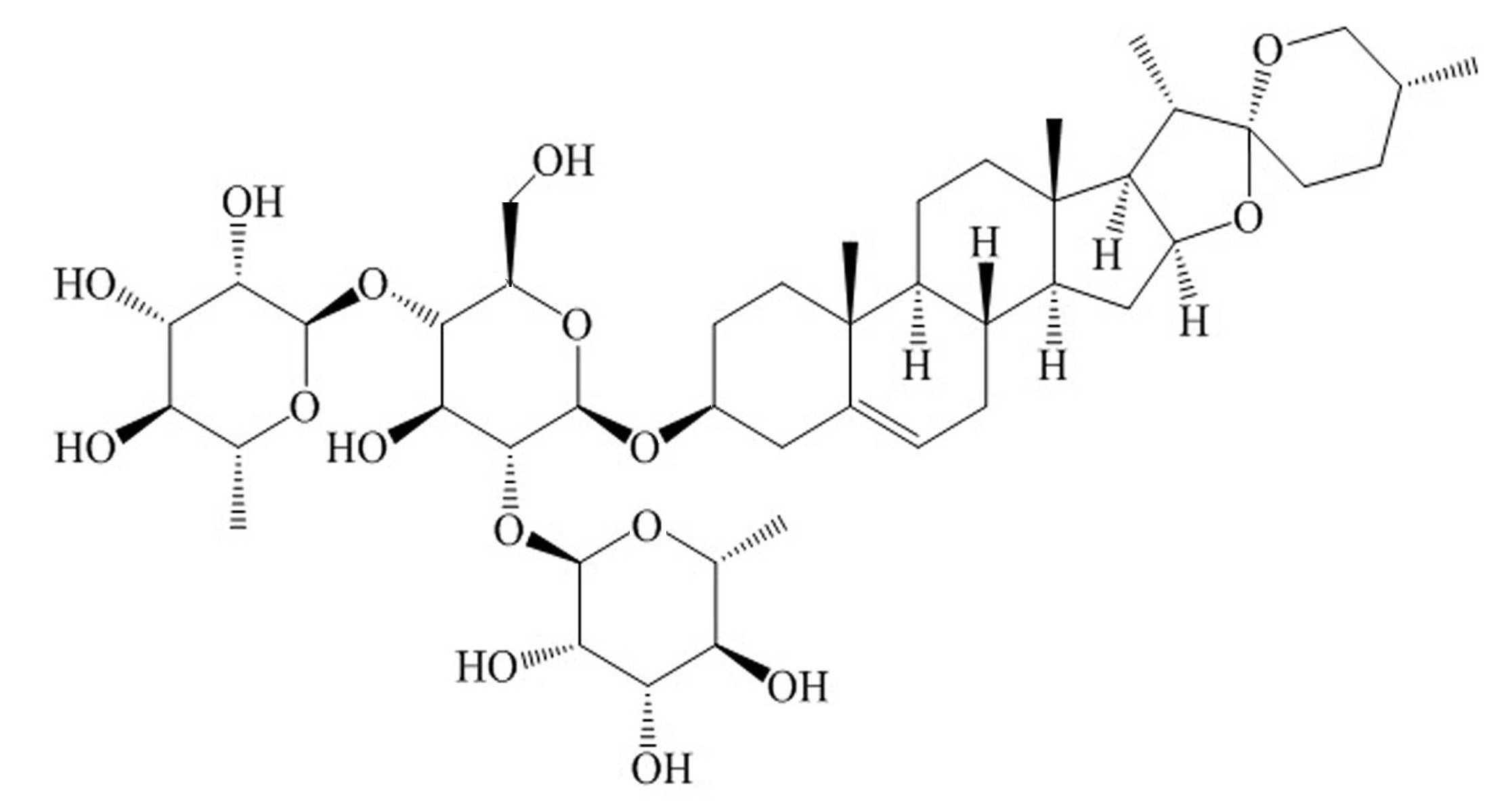


**Supplemental Figure 1**. The chemical structure of dioscin.


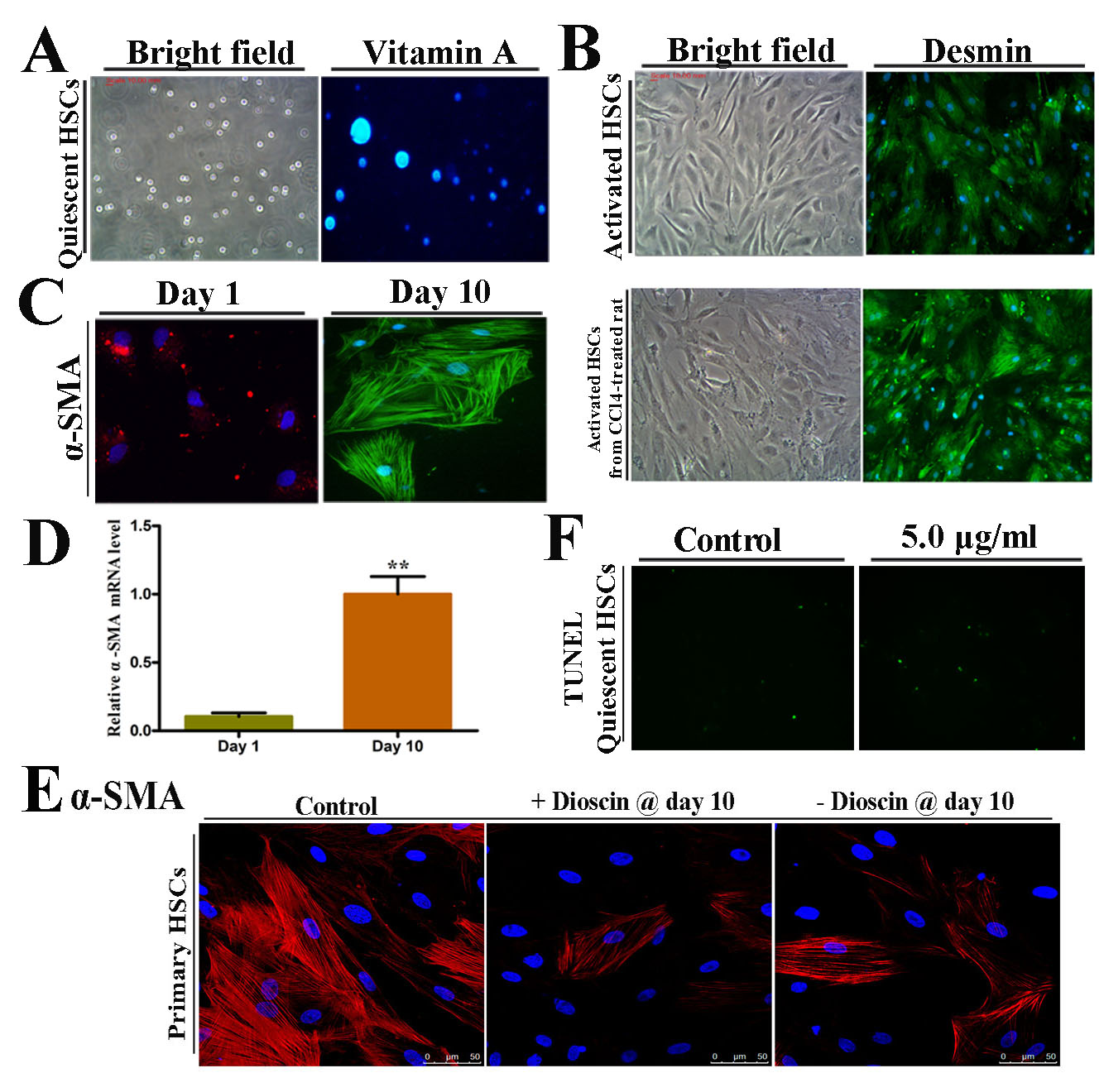


**Supplemental Figure 2.** Identification of primary rat HSCs. (A) Bright field and vitamin A of the quiescent HSCs (100 **×** magnification). (B) Bright field and desmin of the activated HSCs from healthy or CCl_4_-treated rats (100 **×** magnification). (C-D) The expression of α-SMA of the primary rat HSCs evaluated by immunofluorescence and Real-time PCR assays in Day1 and Day10 culture (10000 **×** magnification). (E) Determination of α-SMA expression from primary rat HSCs: freshly isolated rat HSCs were exposed to 1.25 μg/ml dioscin from Day0 to Day10 (+ dioscin @ Day10), then at which time dioscin was washed away and these cells were incubated with complete medium until Day13 (-dioscin @ day 10) (10000 x magnification). (F) Day-2 cultured HSCs were incubated with 5.0 μg/ml dioscin for 24 h, and TUNEL staining was performed (100 **×** magnification). Data are presented as the mean ± SD (n = 3). *p < 0.05 and **p < 0.01 compared with the Day 1 group.


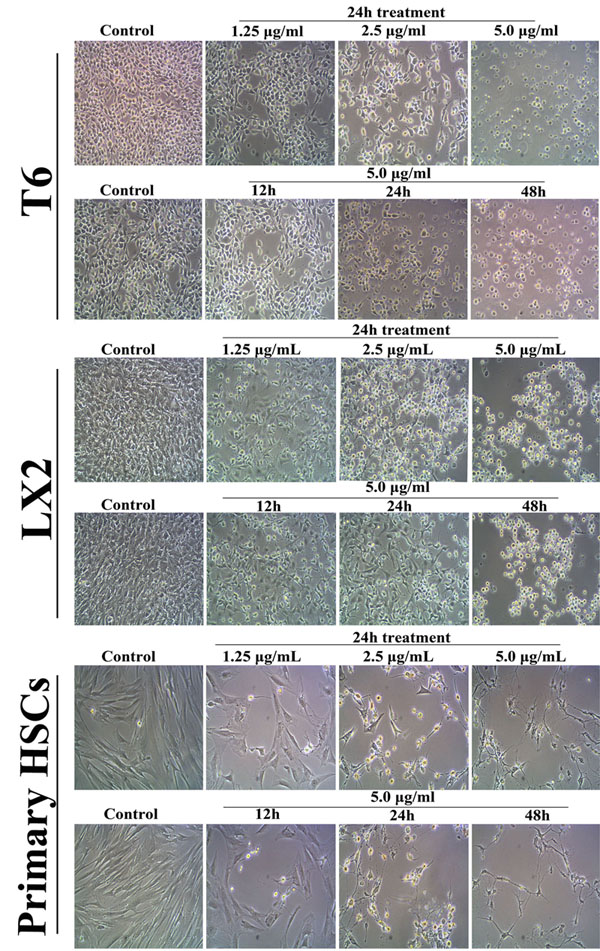


**Supplemental Figure 3.** The bright field of HSC-T6, LX2 and primary HSCs treated by different concentrations of dioscin (1.25, 2.5 and 5.0 μg/ml) under different treatment times (12, 24 and 48 h). (40 × magnification)

**
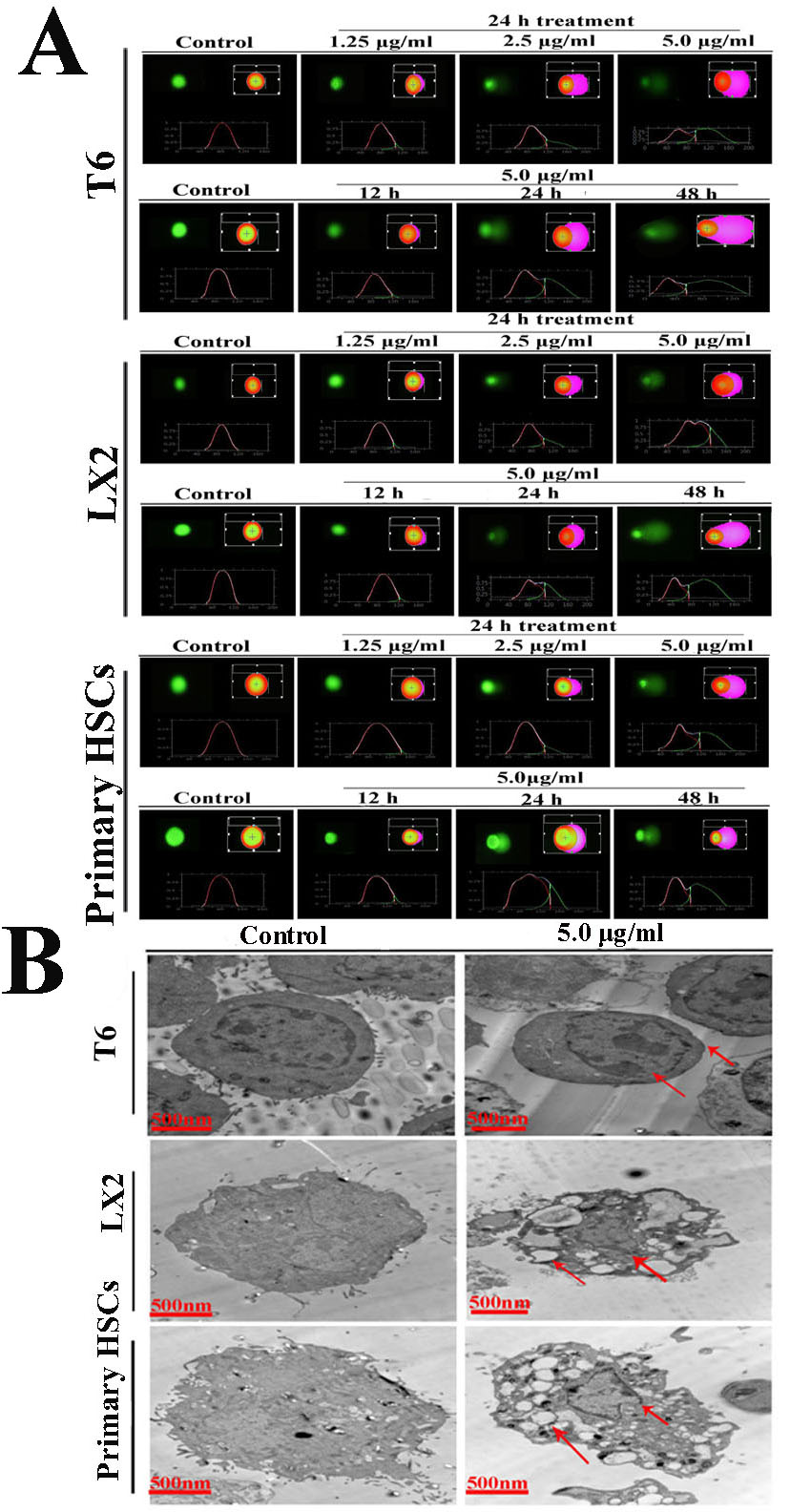
**

**Supplemental Figure 4.** Dioscin induced HSC apoptosis in HSC-T6, LX2 and primary HSCs. (A) Dioscin-induced DNA damage (200 ×, final magnification) based on the SCGE assay. (B) Dioscin induced changes in cell ultra-structures based on TEM assay.

**
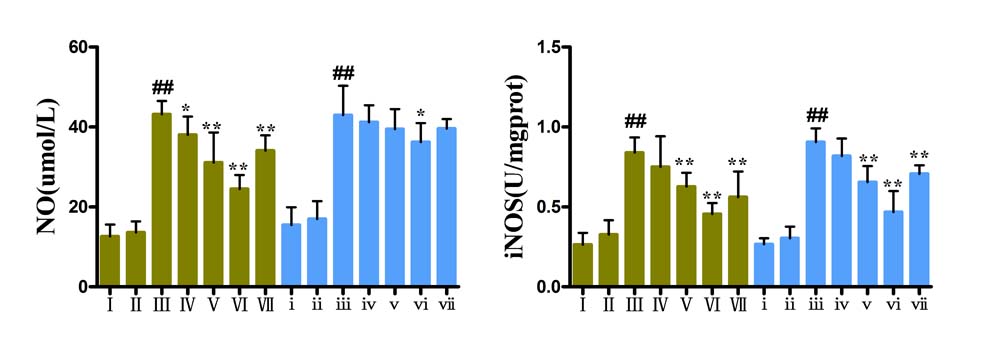
**

**Supplemental Figure 5.** Effects of dioscin on the levels of NO and iNOS. Results are expressed as mean ± SD. *p < 0.05, **p < 0.01 vs. model group; ^##^p < 0.01 vs. normal control group (n = 10).

**
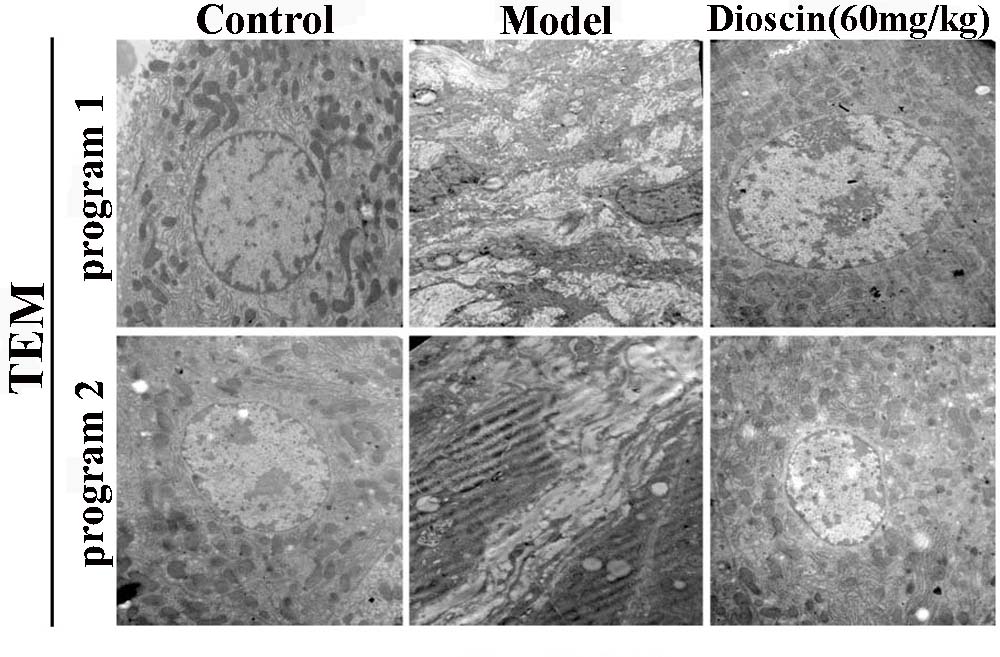
**

**Supplemental Figure 6.** Effects of dioscin against CCl_4_-induced liver fibrosis in rats based on TEM assay.


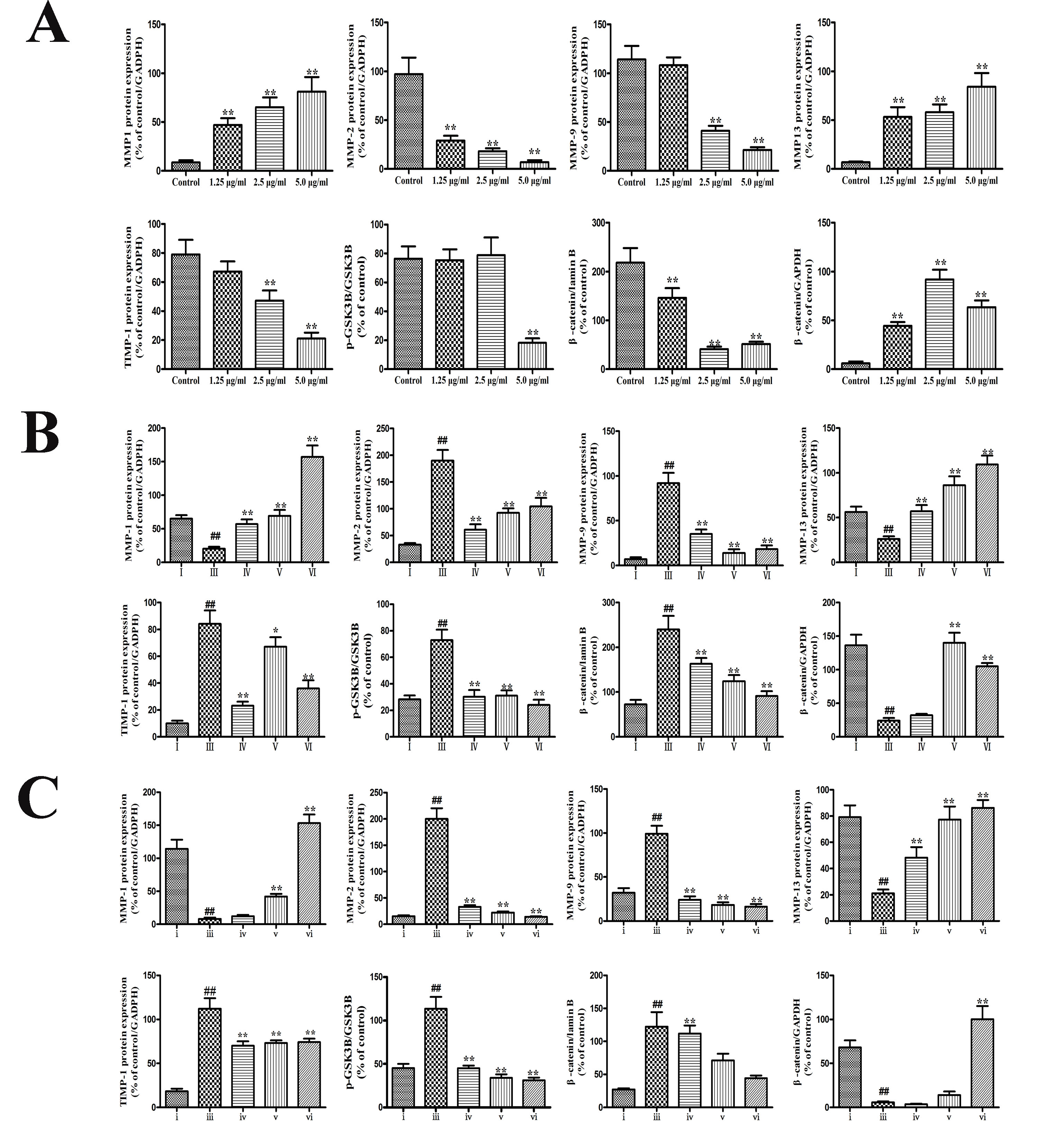


**Supplemental Figure 7.** Effects of dioscin on ECM accumulation and Wnt/β-catenin signaling *in vitro* and *in vivo*. (A) Primary rat HSCs from healthy rats were treated with various concentrations of dioscin for 24 h. Western blot analysis of MMP-1, MMP-2, MMP-9, MMP-13, TIMP-1, p-GSK3β/GSK3β, β-catenin/lamin B, β-catenin/GAPDH. (B) Western blot analysis of MMP-1, MMP-2, MMP-9, MMP-13, TIMP-1, p-GSK3β/GSK3β, β-catenin/lamin B, β-catenin/GAPDH in program I. (C) Western blot analysis of MMP- 1, MMP-2, MMP-9, MMP-13, TIMP-1, p-GSK3β/GSK3β, β-catenin/lamin B, β-catenin/ GAPDH in program II.


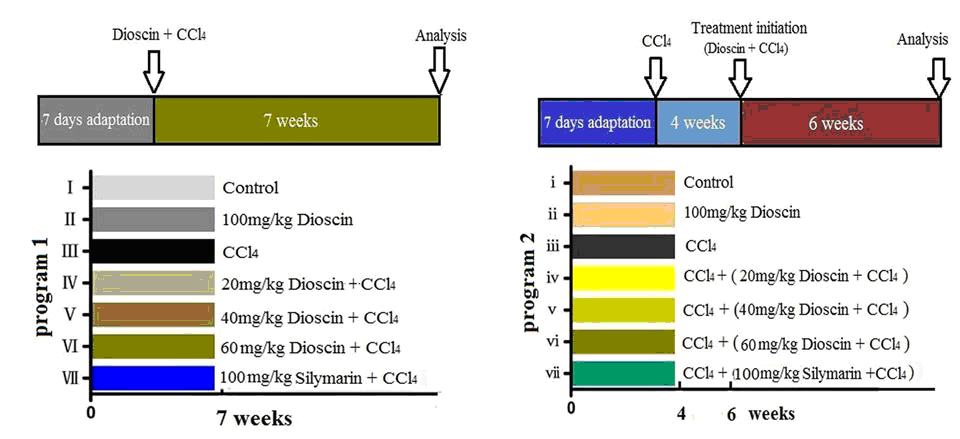


**Supplemental Figure 8.** The experimental design. The rats were treated by intraperi -toneal (i.p.) injection of CCl_4_, and dioscin was administered intragastrically (i.g.) to the rats.


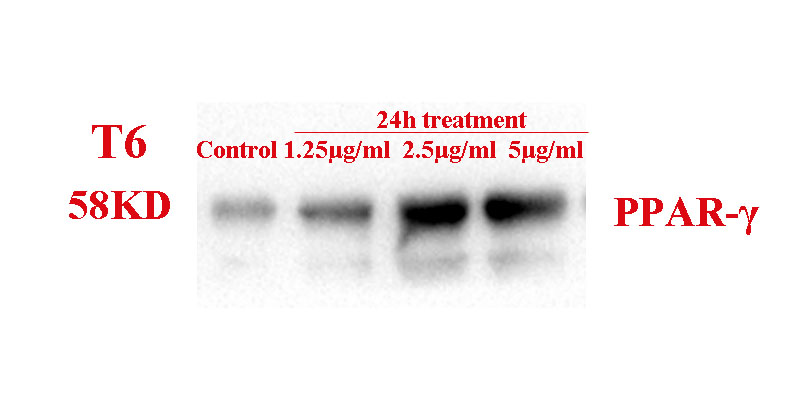


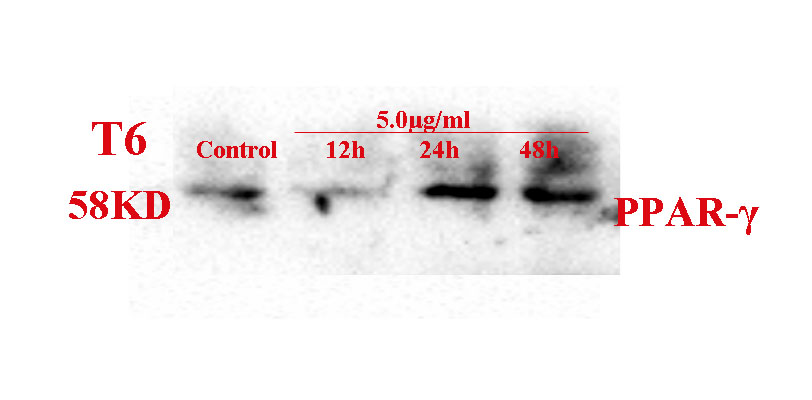


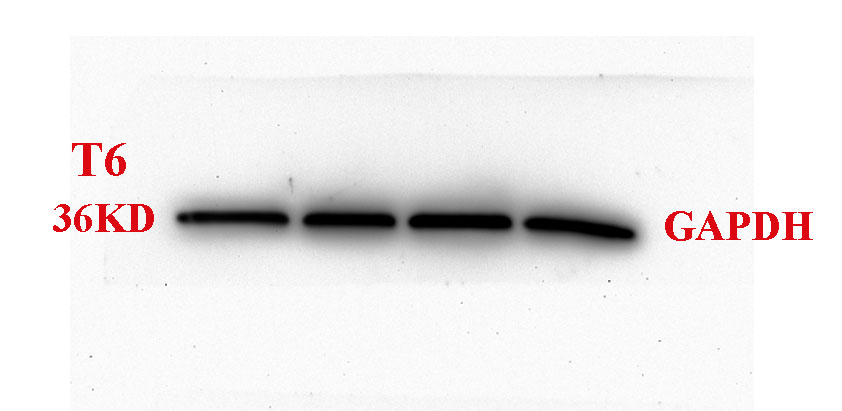


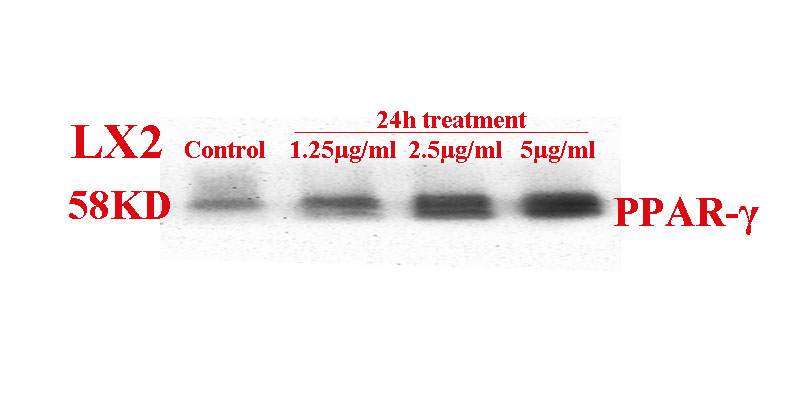


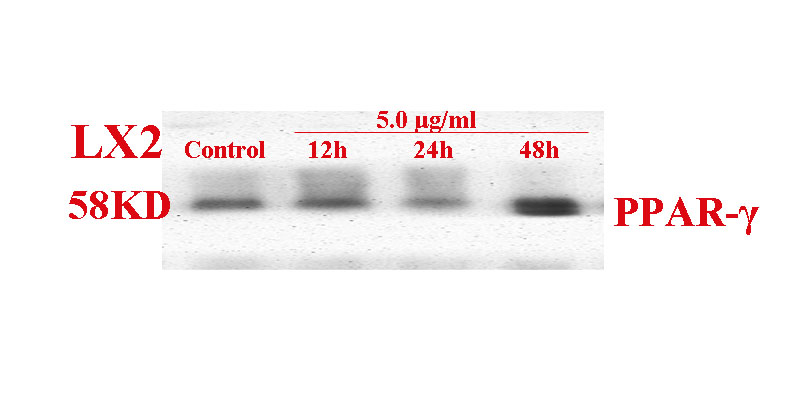


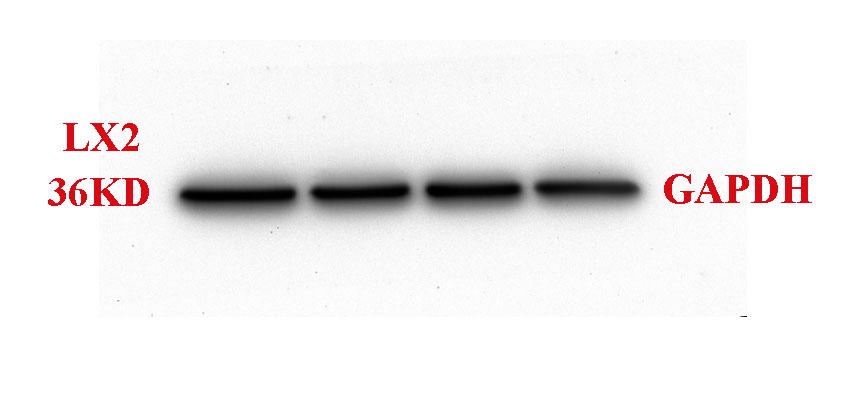


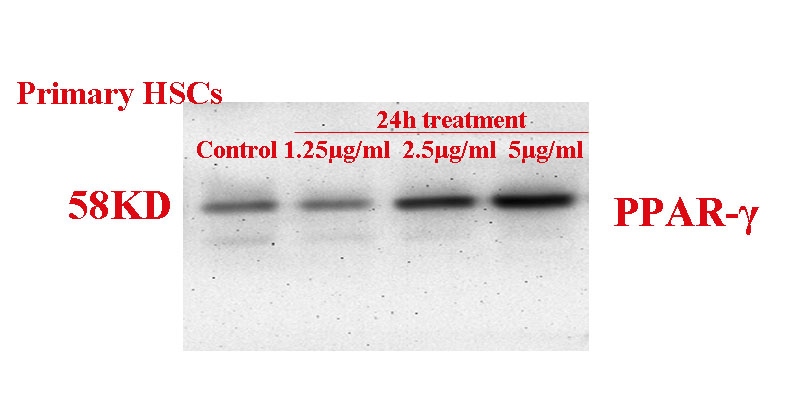


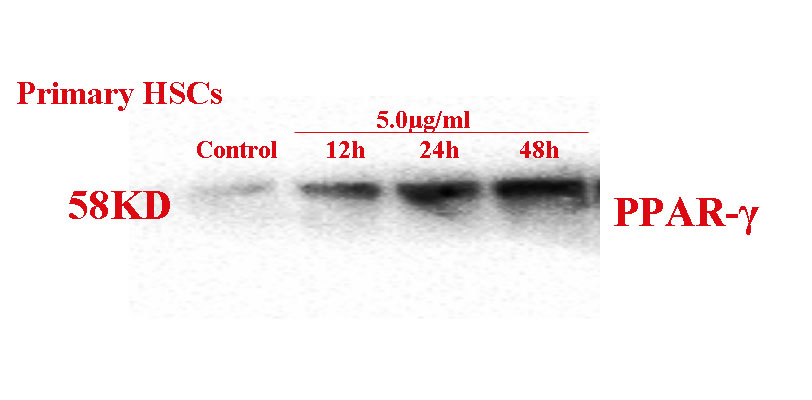


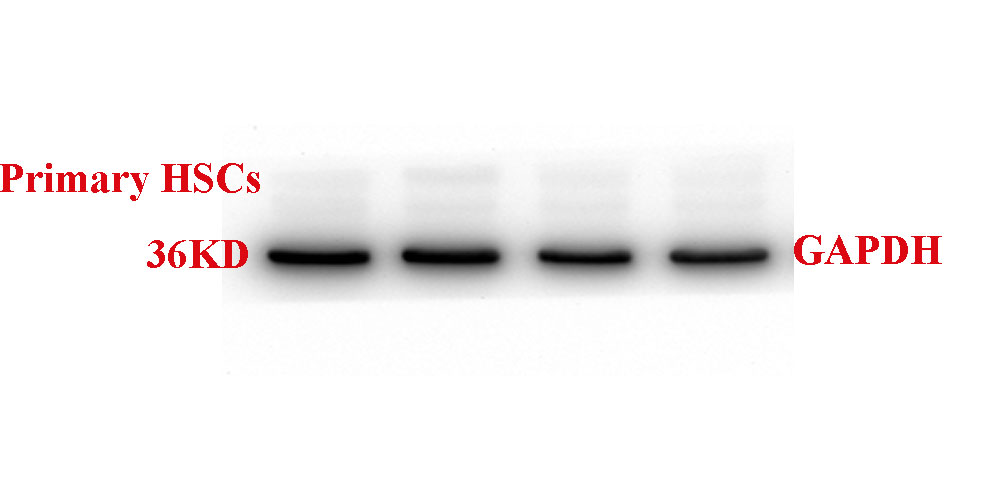


**Supplemental Figure 9.** Effects of dioscin on the protein expression levels of PPAR-γ in HSC-T6, LX2 and primary HSCs.


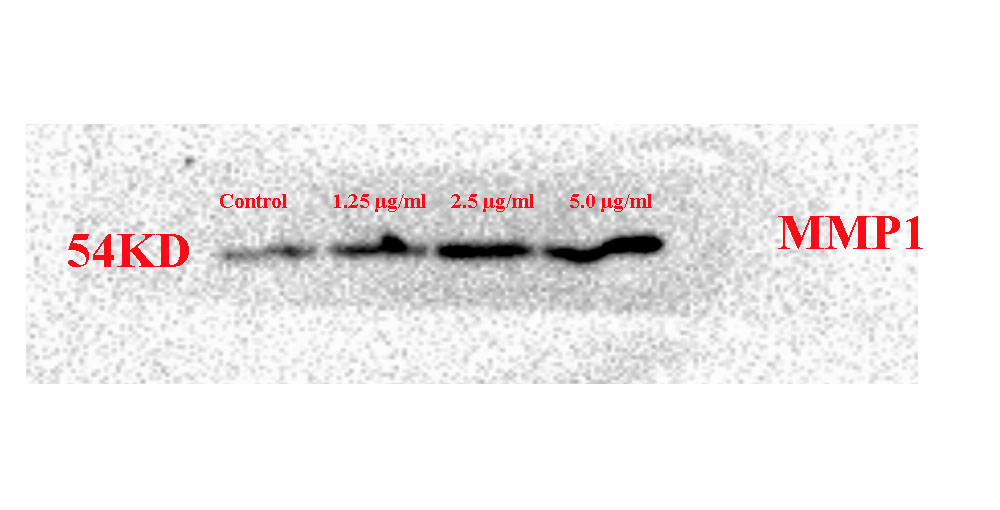


**
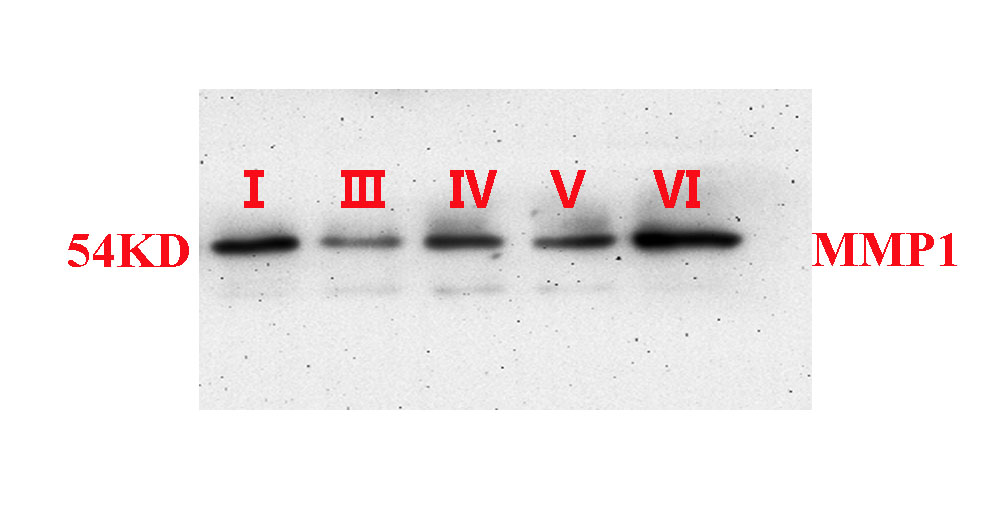
**

**
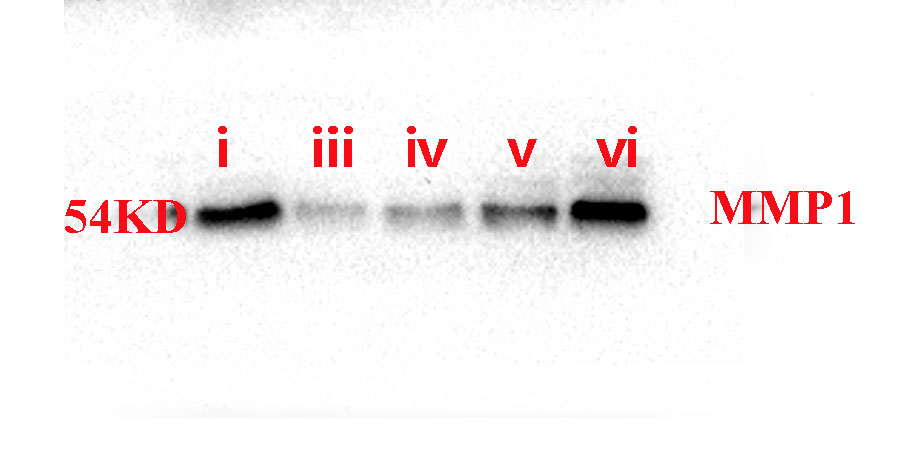
**

**
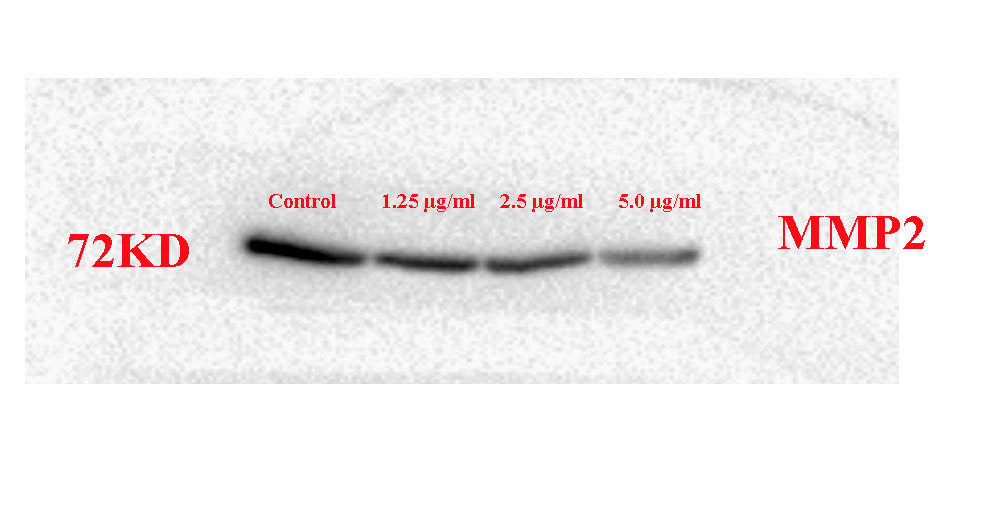
**

**
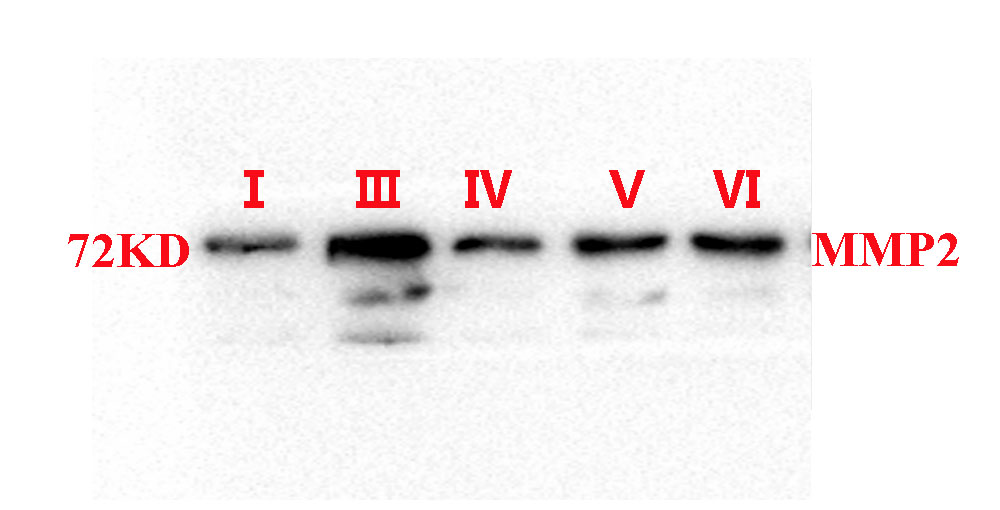
**

**
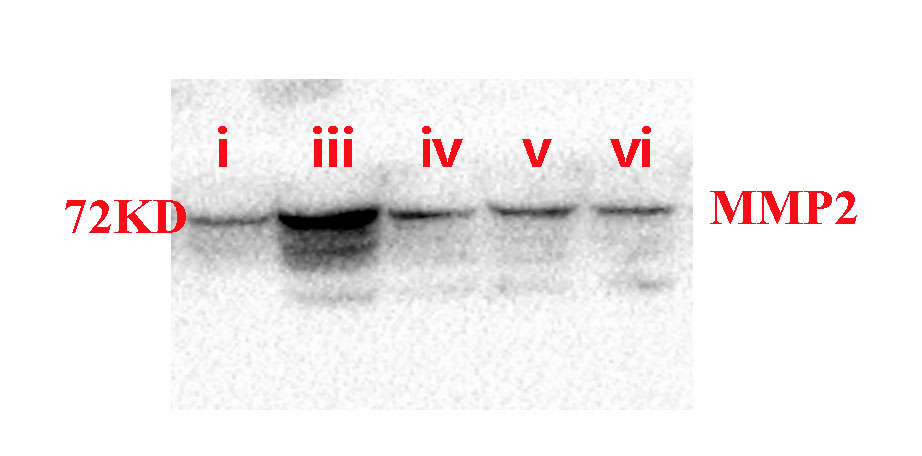
**

**
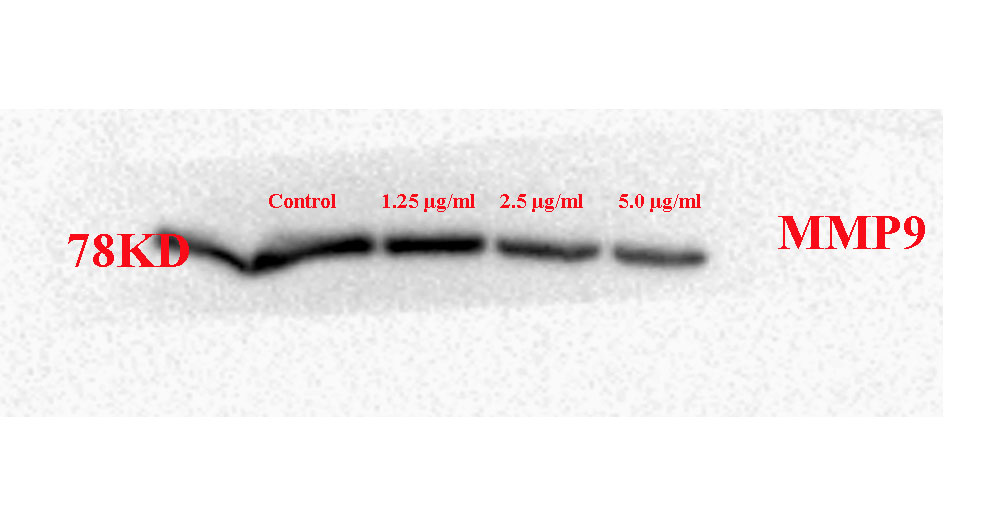
**

**
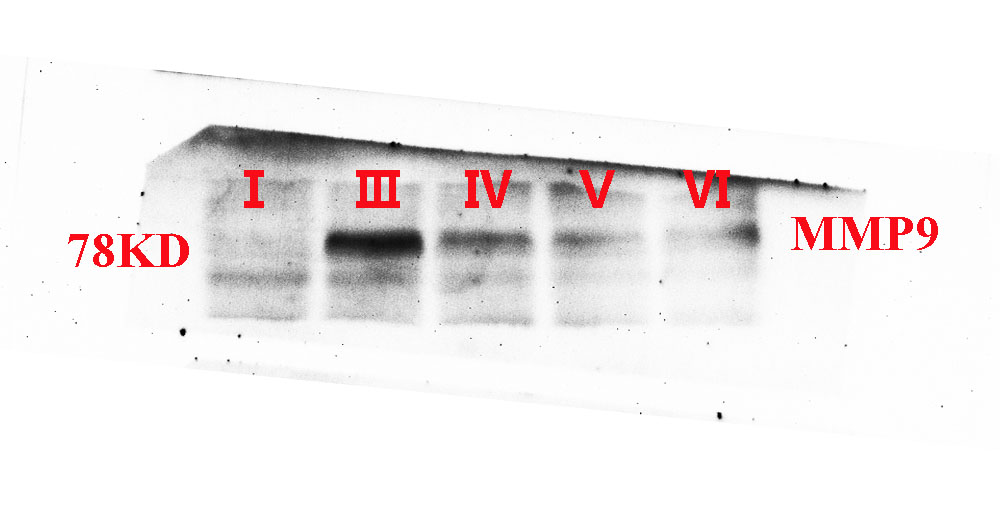
**

**
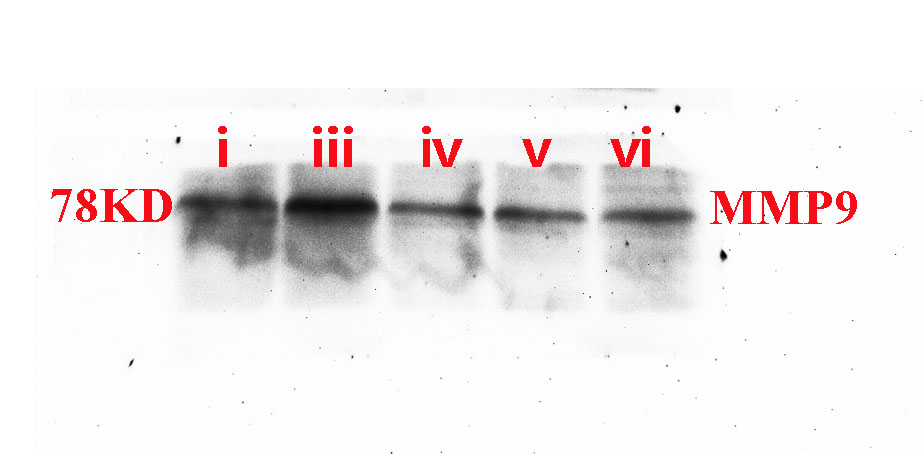
**

**
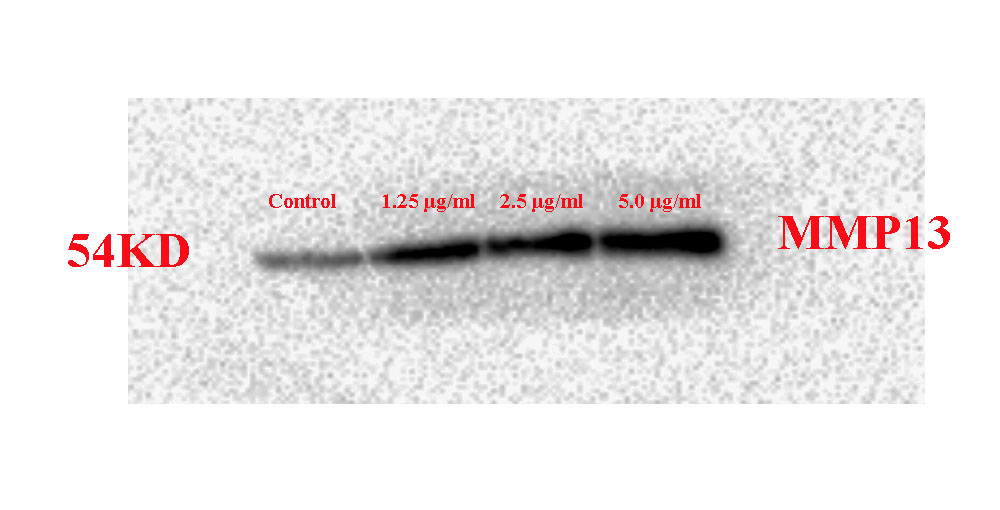
**

**
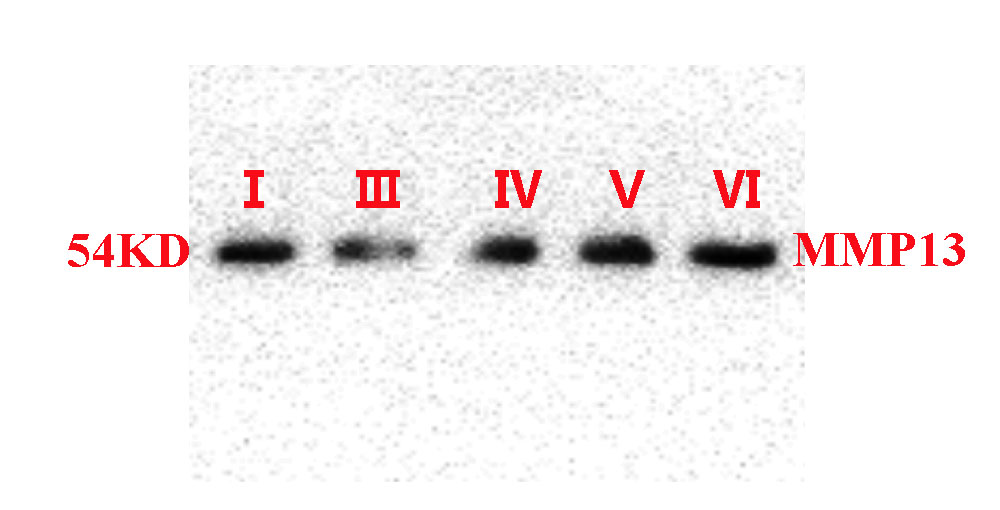
**

**
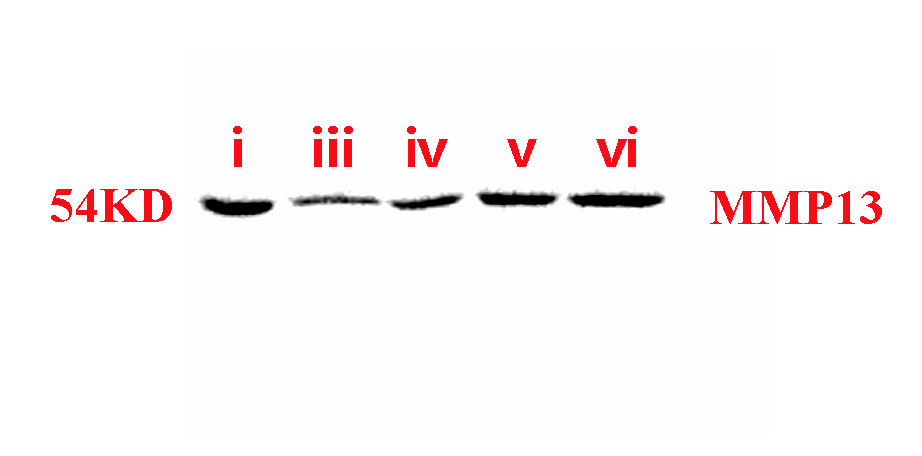
**

**
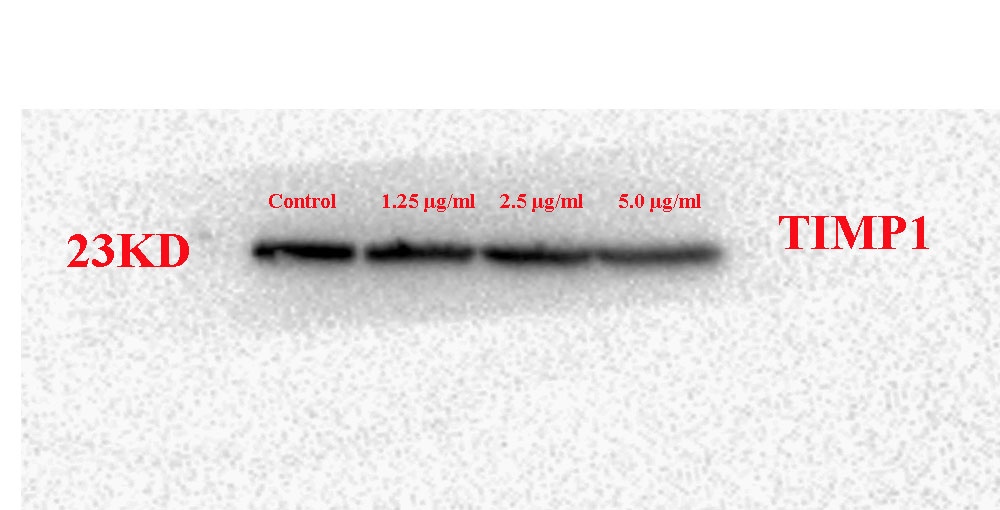
**

**
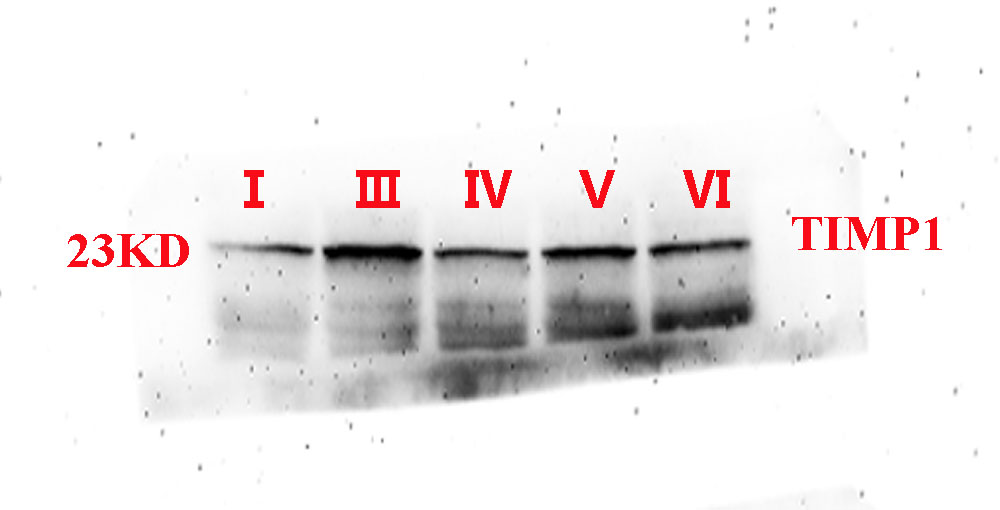
**

**
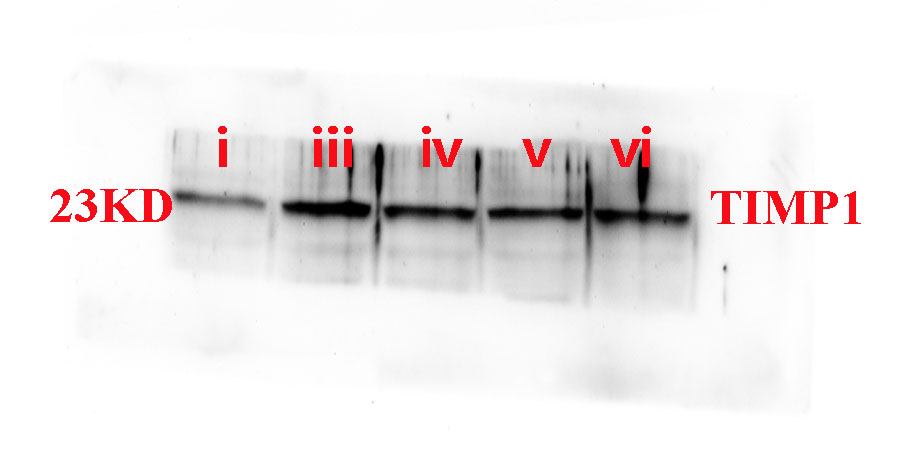
**

**
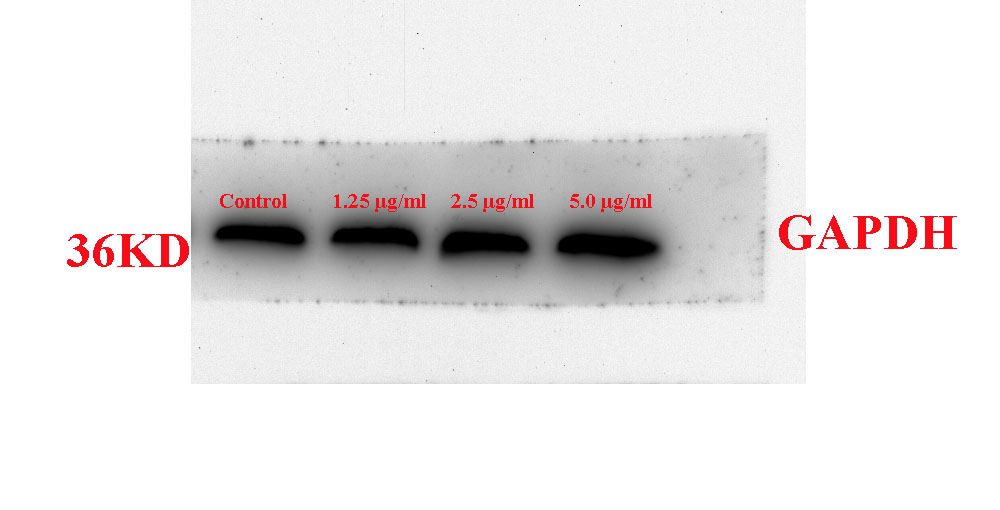
**

**
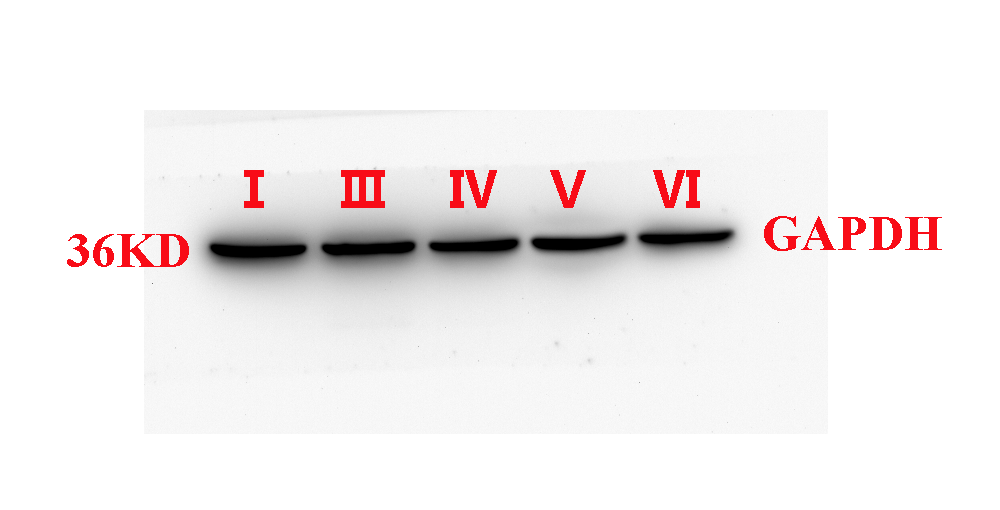
**

**
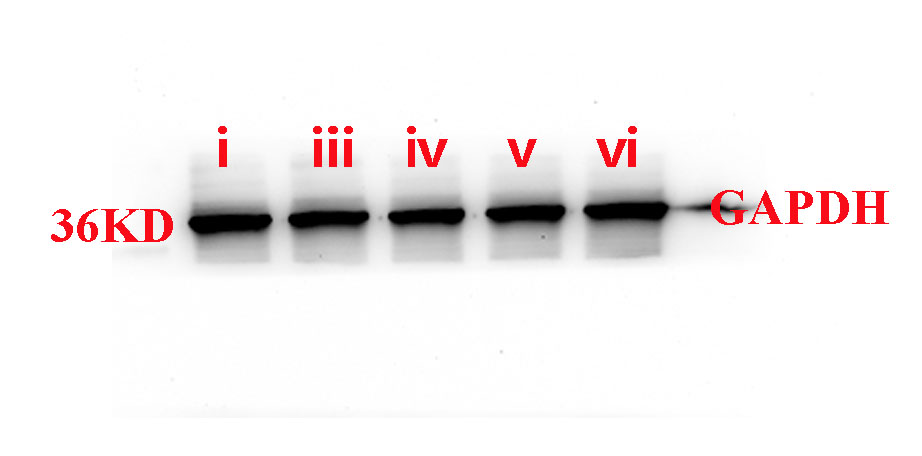
**

**Supplemental Figure 10.** Effects of dioscin on the protein expression levels of MMP1, MMP2, MMP9, MMP13 and TIMP1 *in vivo*.


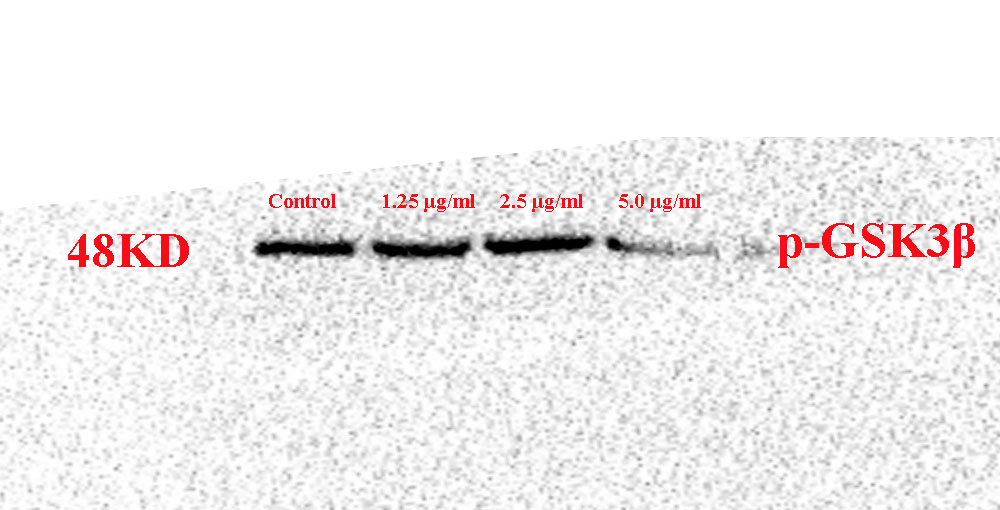


**
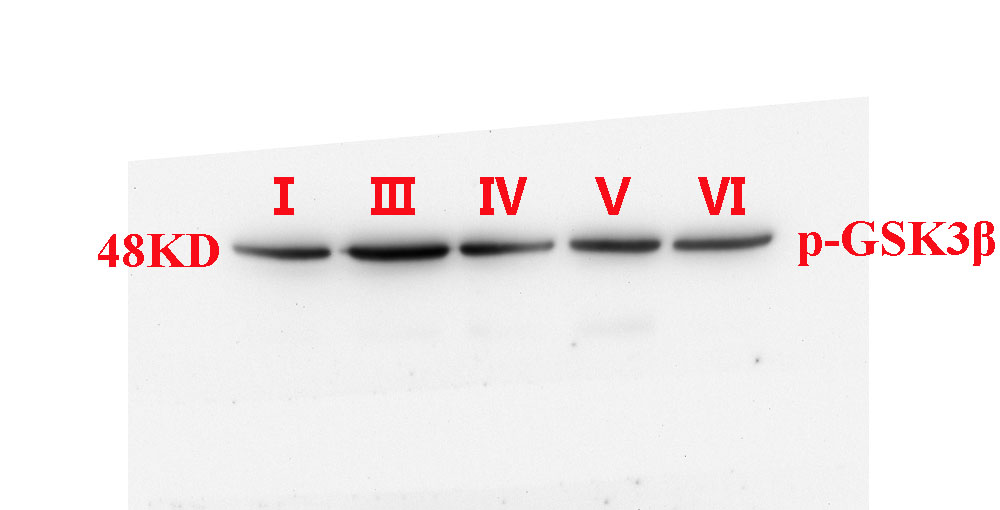
**

**
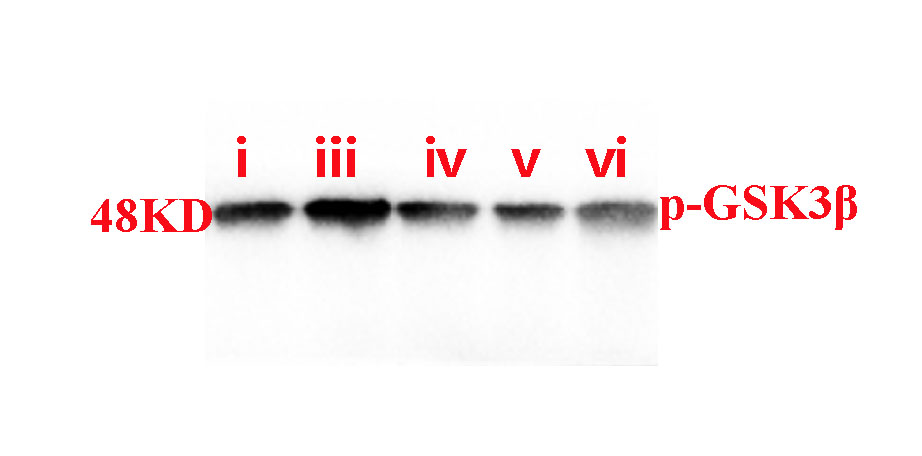
**

**
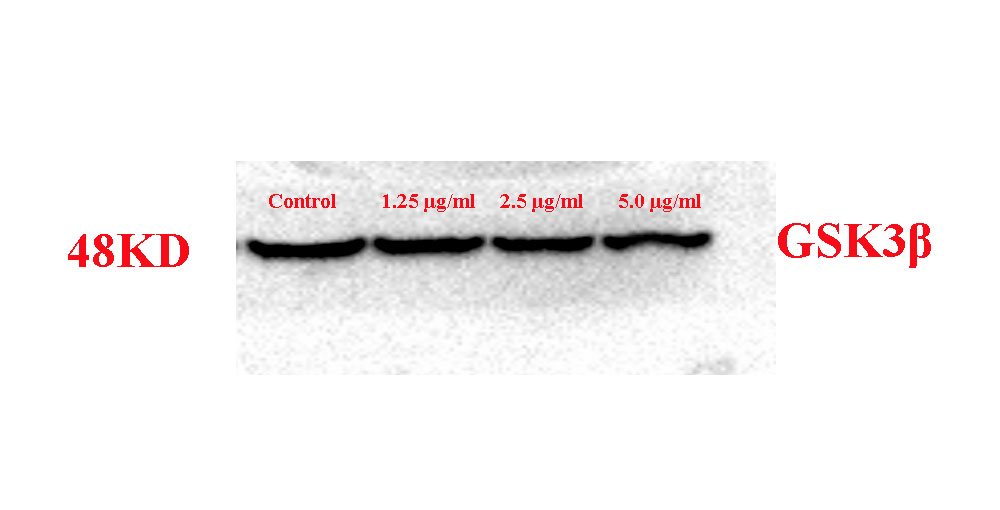
**

**
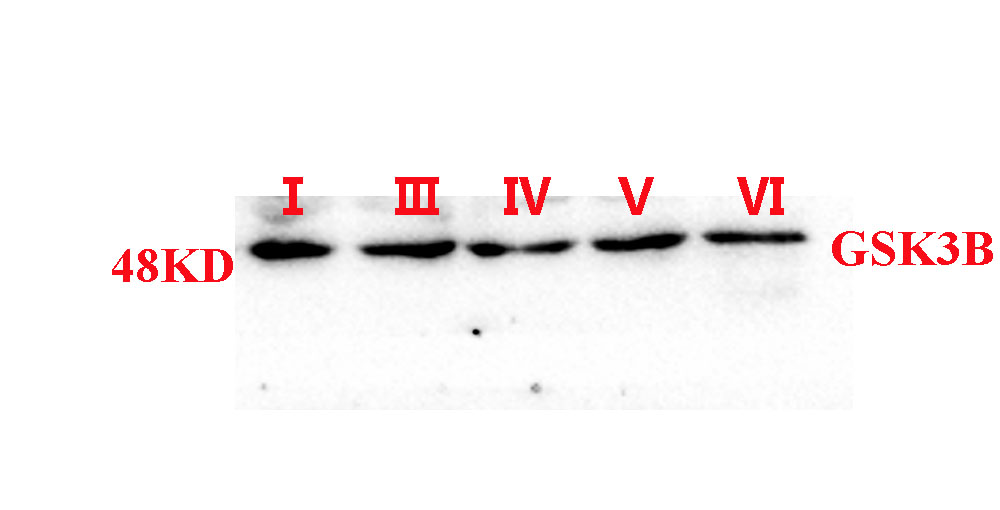

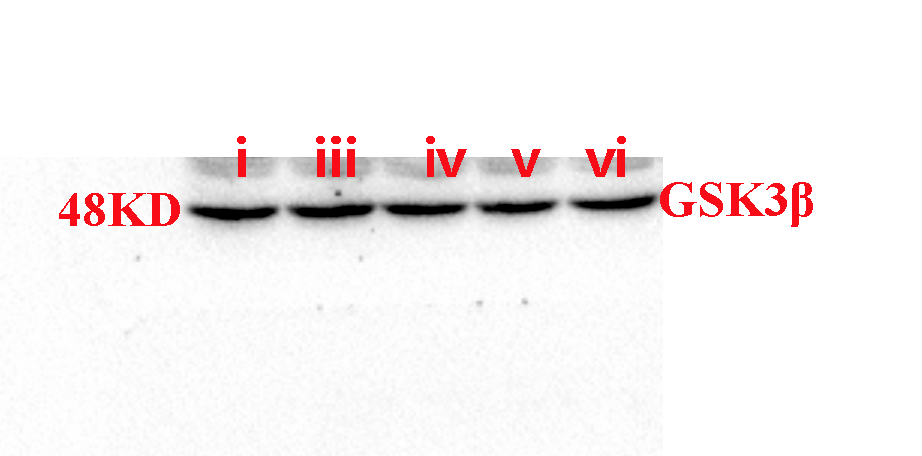
**

**
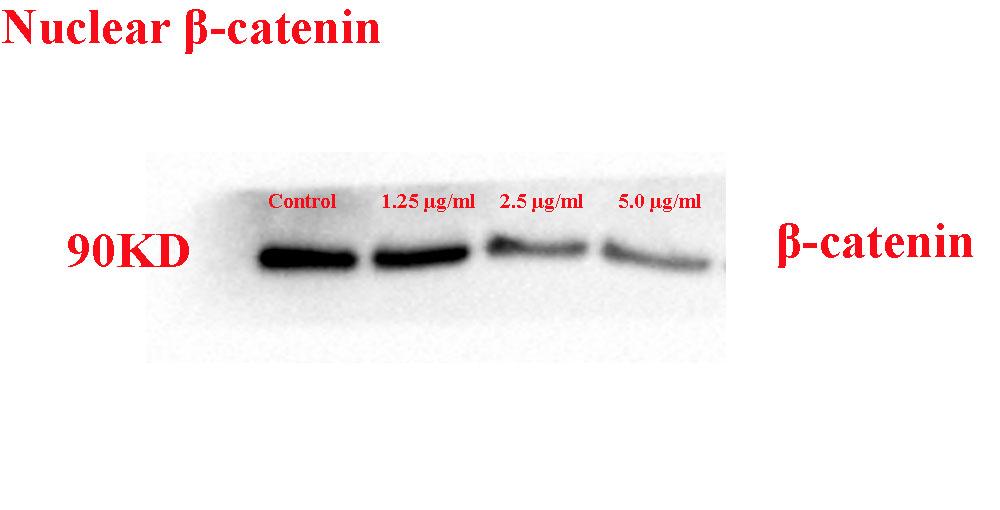
**

**
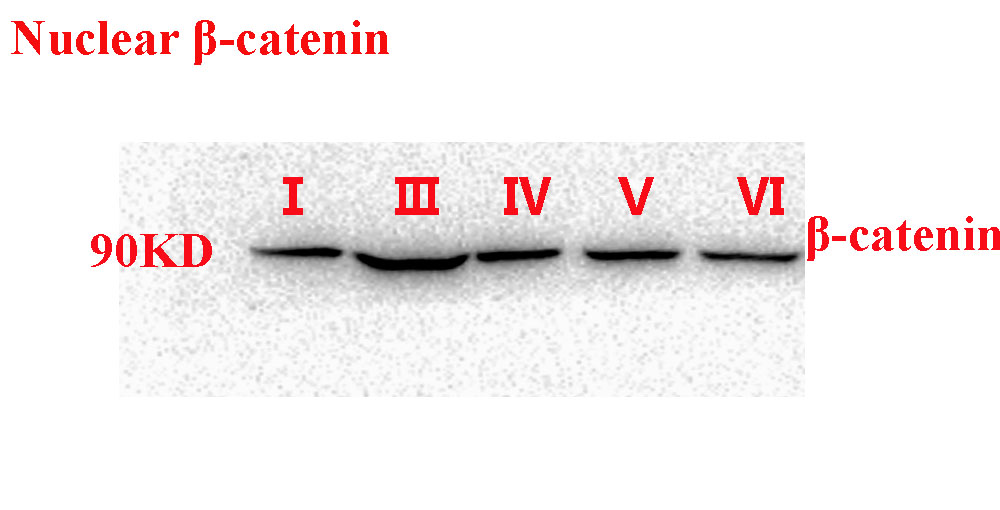
**

**
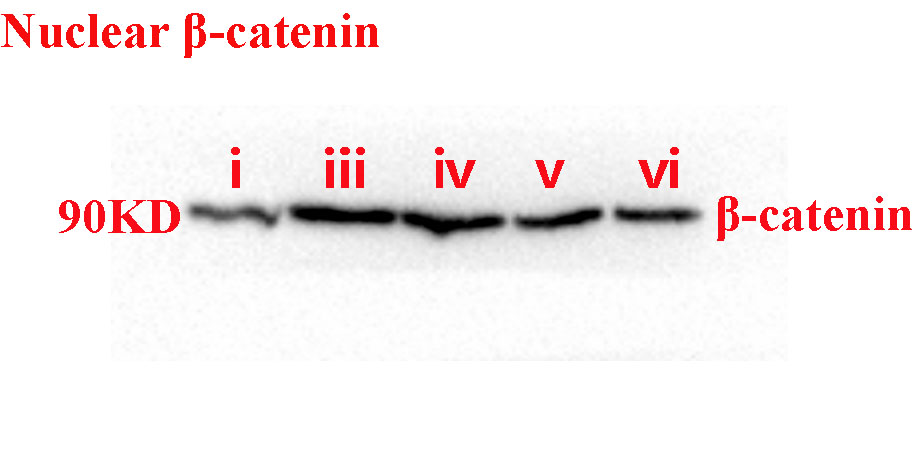
**

**
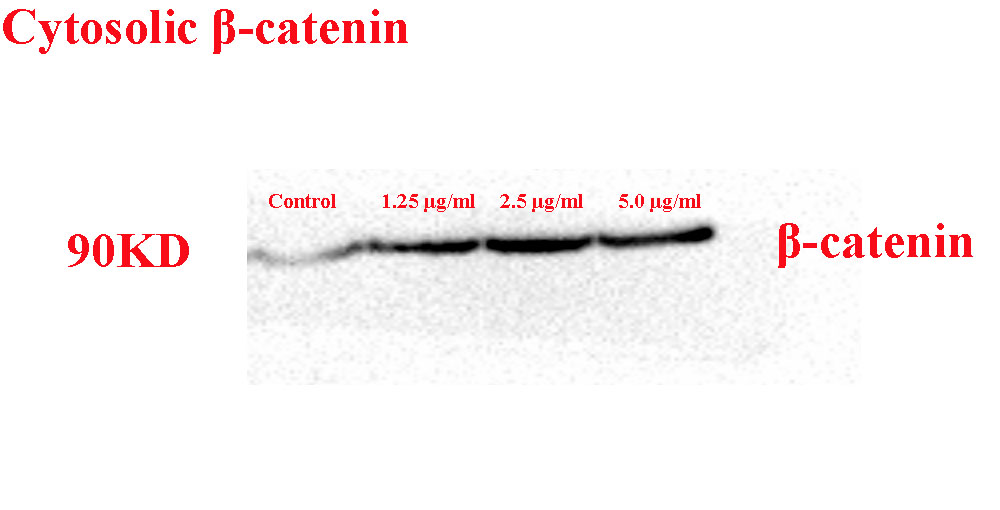

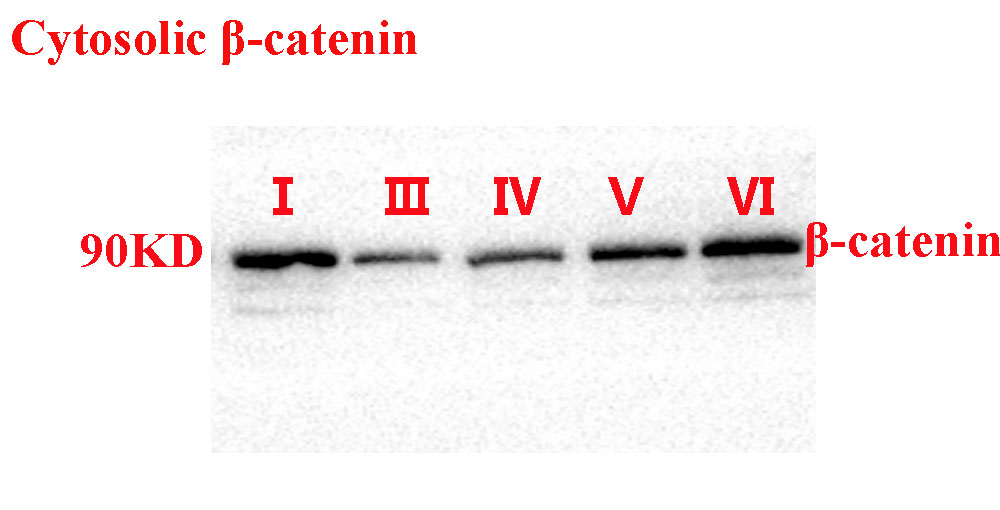

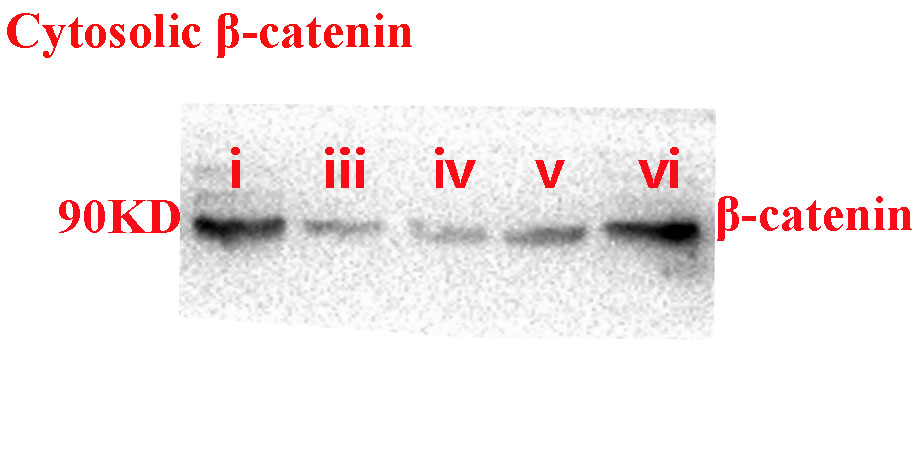
**

**
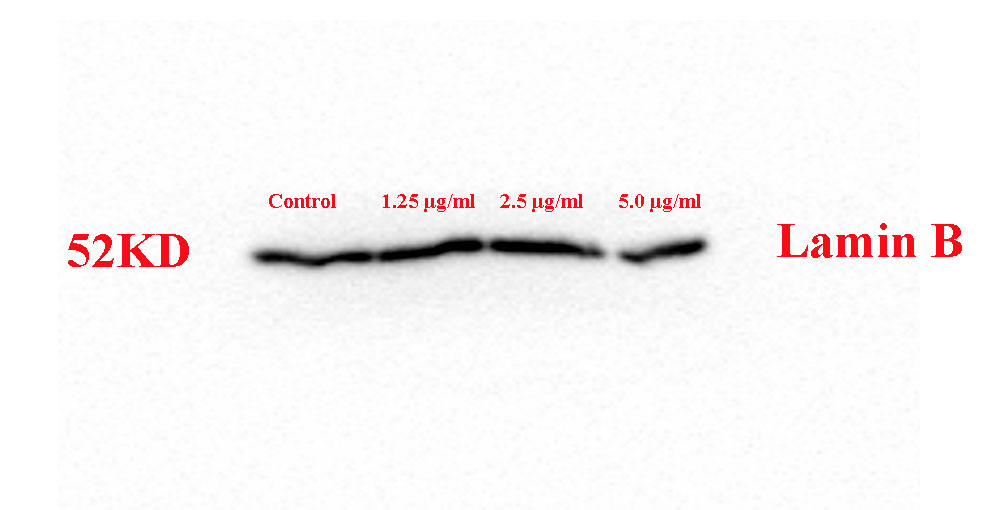
**

**
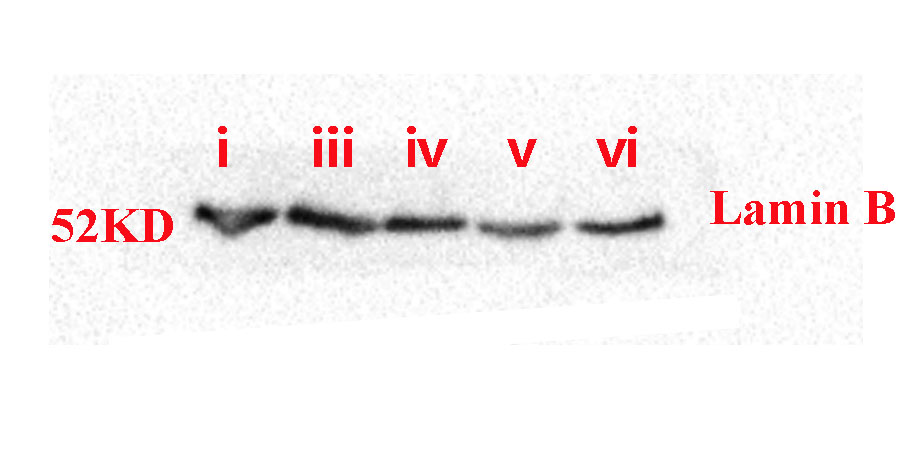

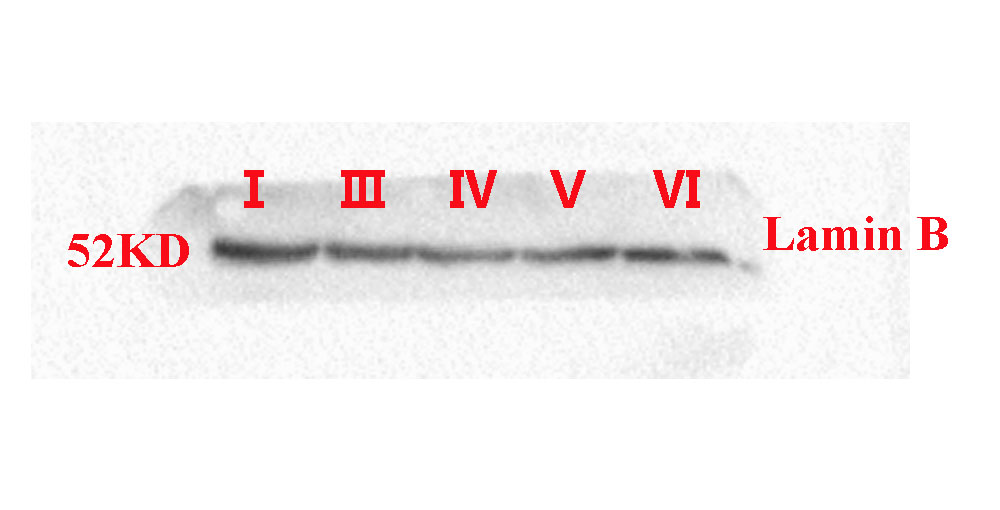
**

**
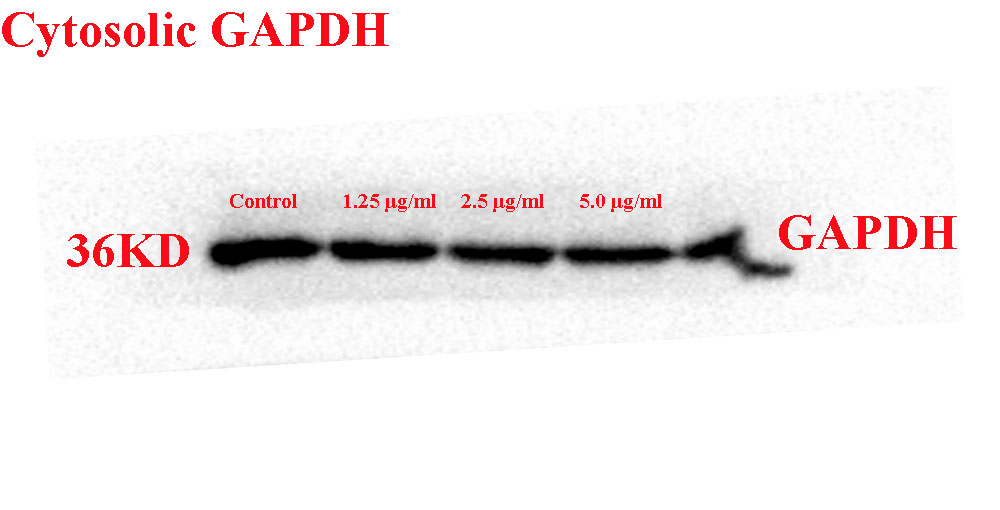
**

**
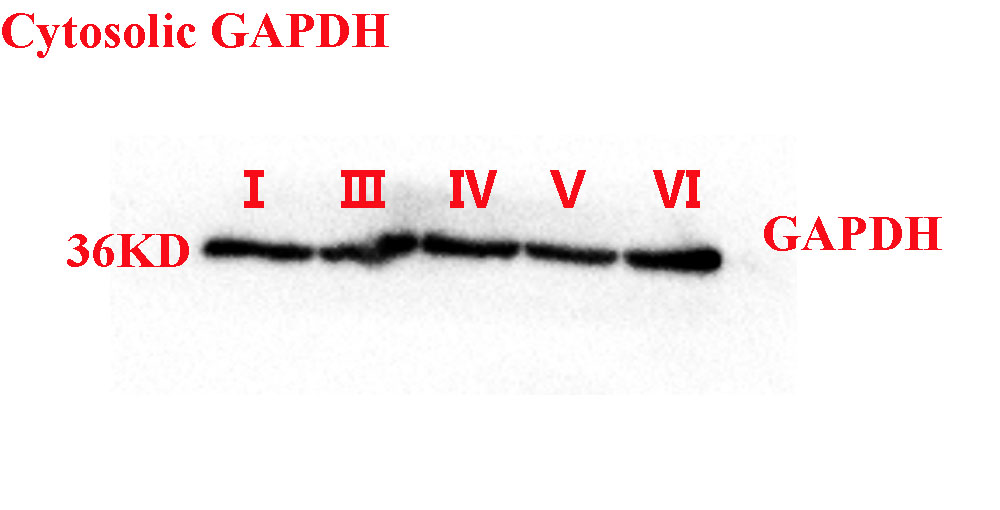
**

**
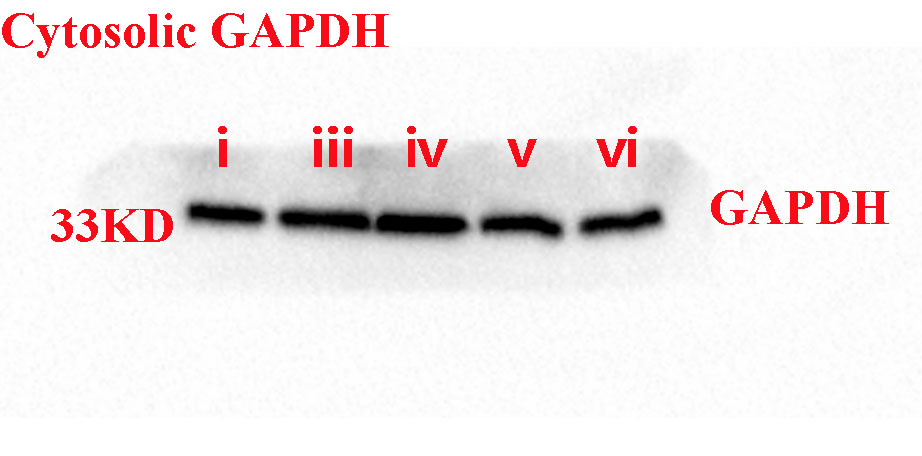
**

**
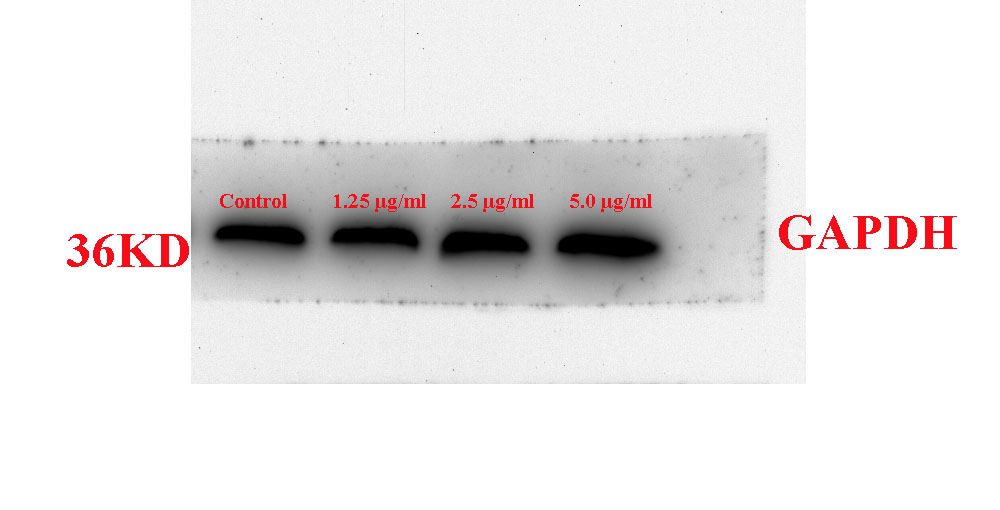
**

**
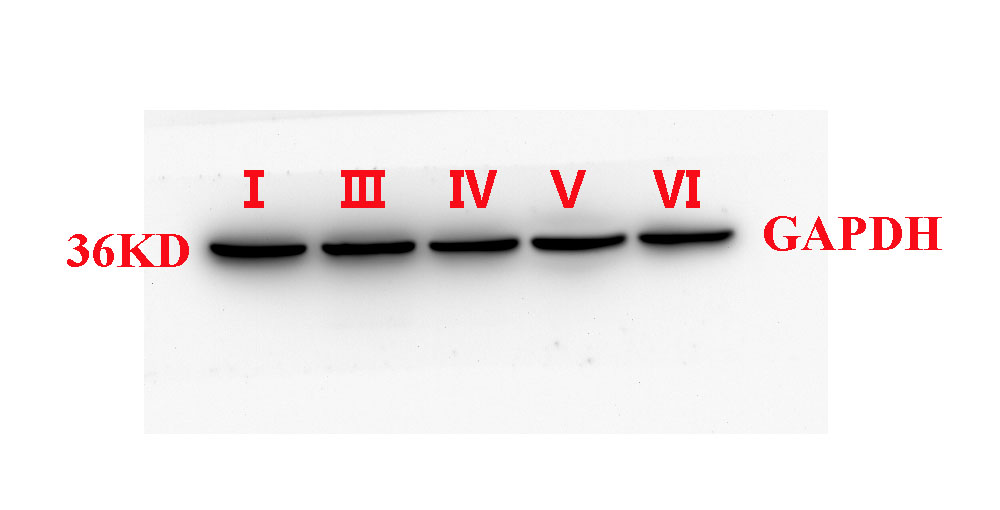
**

**
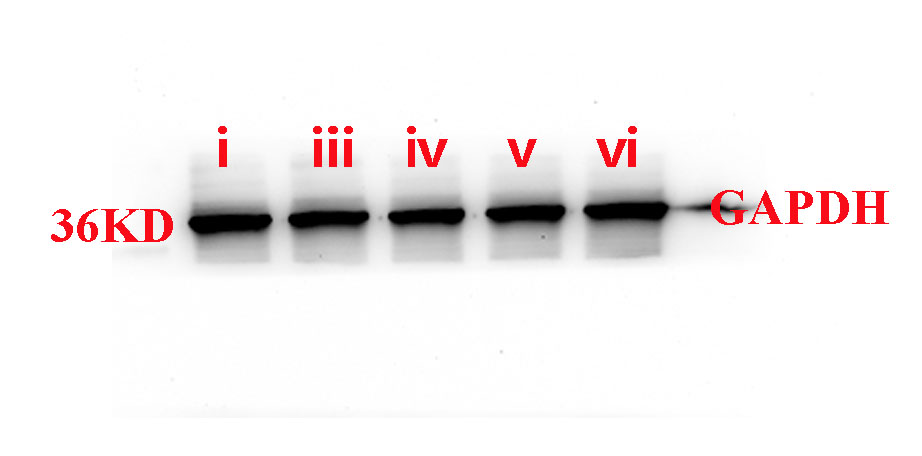
**

**Supplemental Figure 11.** Effects of dioscin on the protein expression levels of p-GSK3β, GSK3β and β-catenin *in vivo*.

**
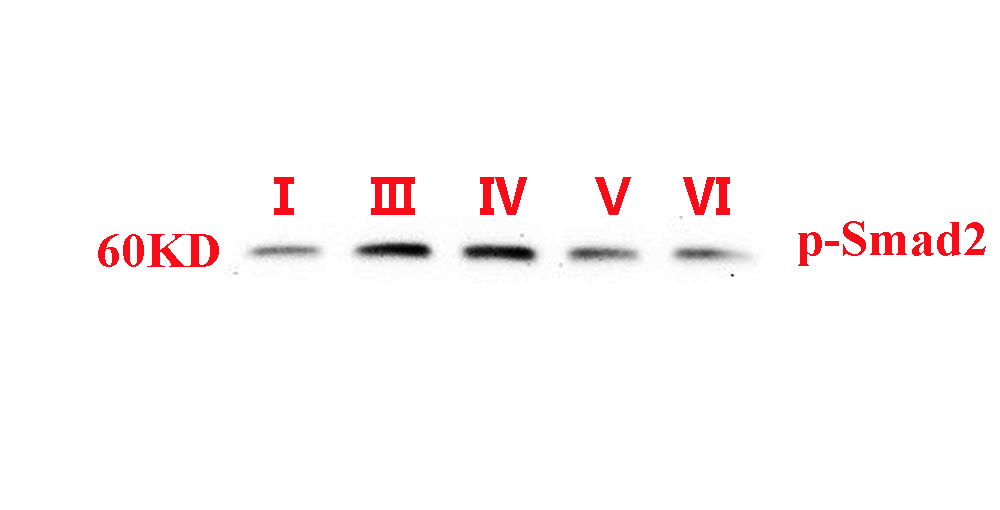
**

**
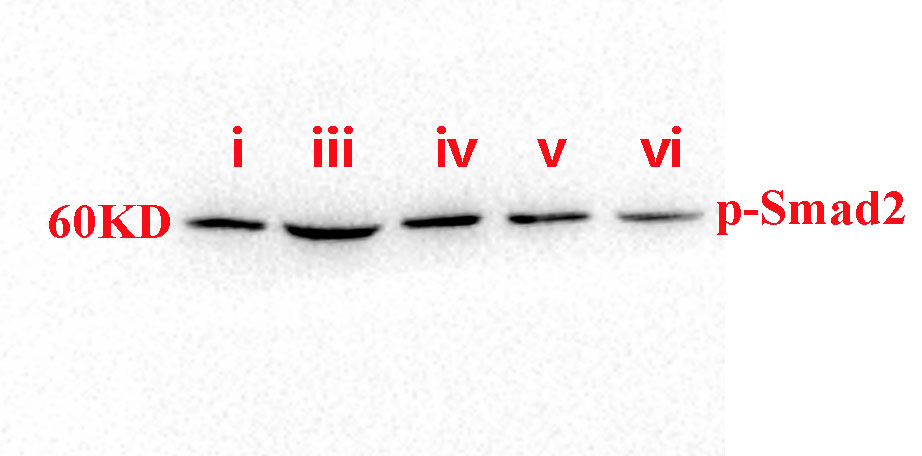
**

**
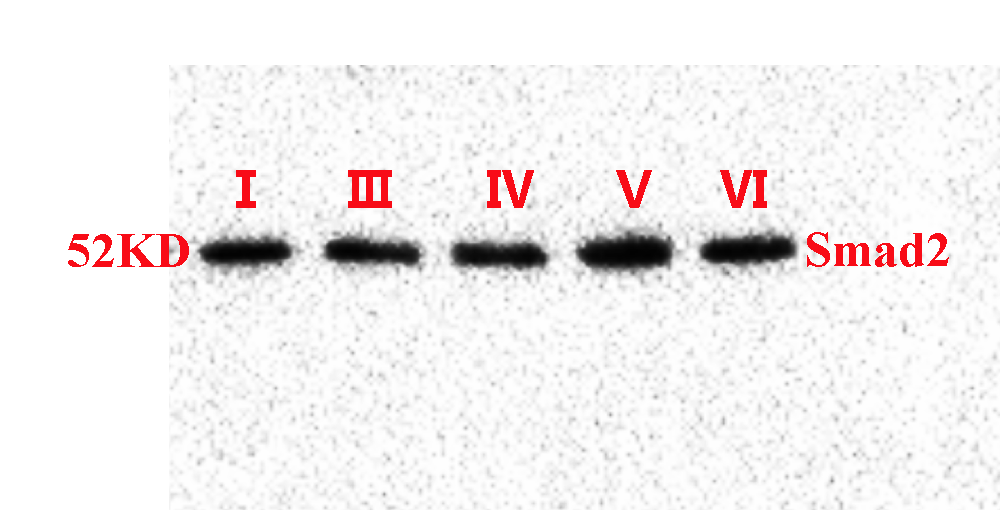
**

**
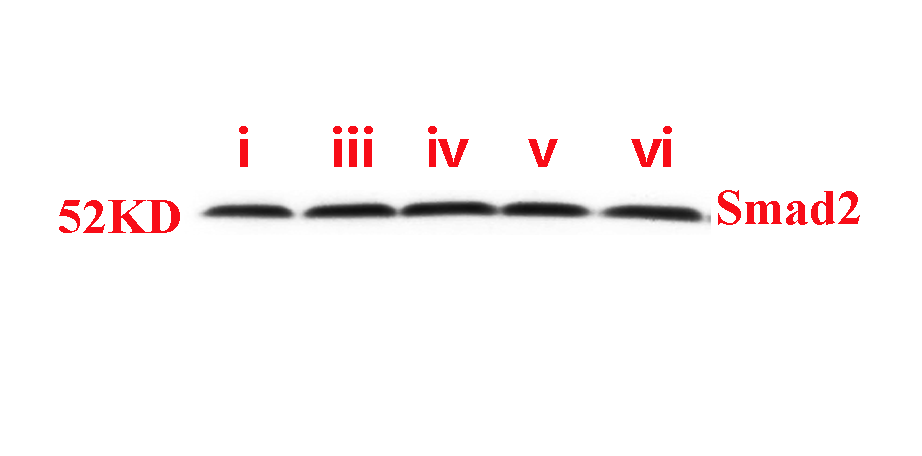
**

**
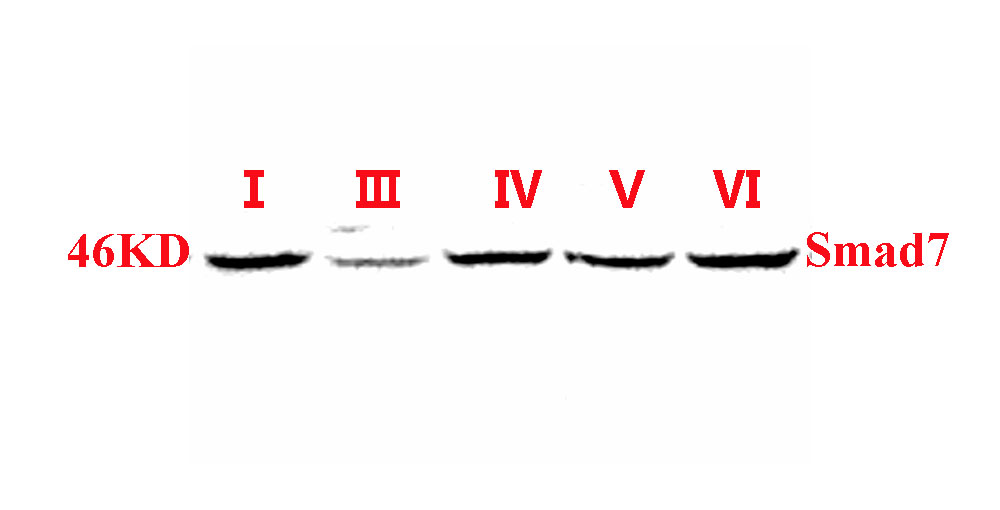
**

**
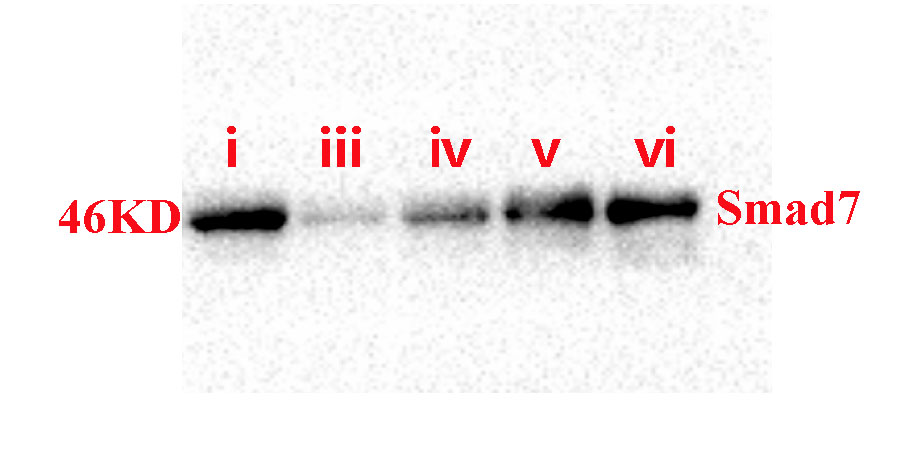
**

**
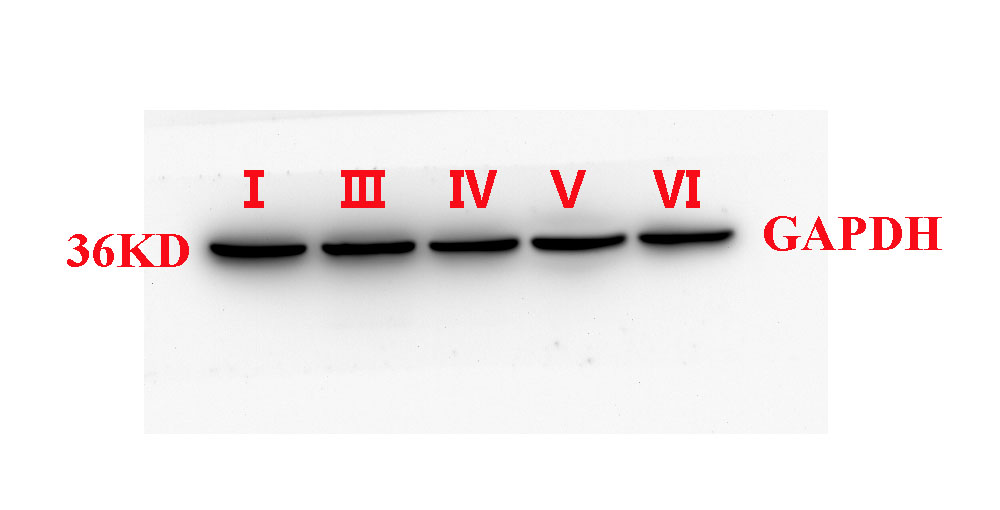
**

**
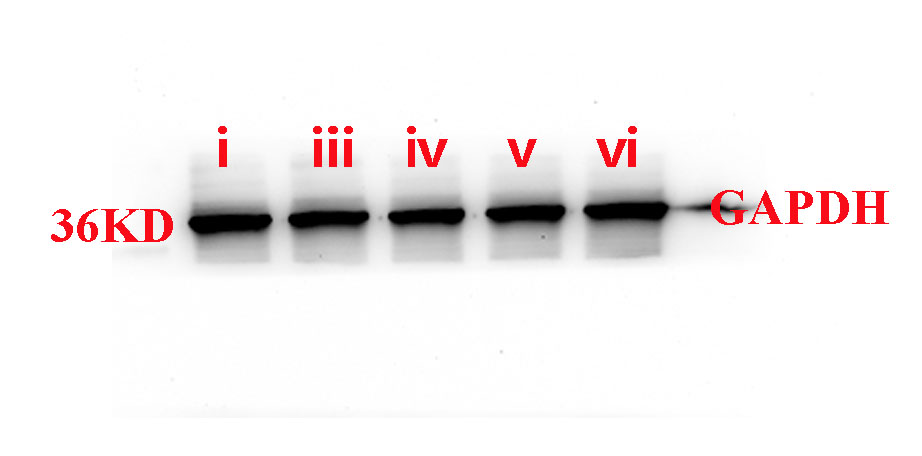
**

**Supplemental Figure 12.** Effects of dioscin on the protein expression levels of p- Smad2, Smad2 and Smad7 *in vivo*.

**
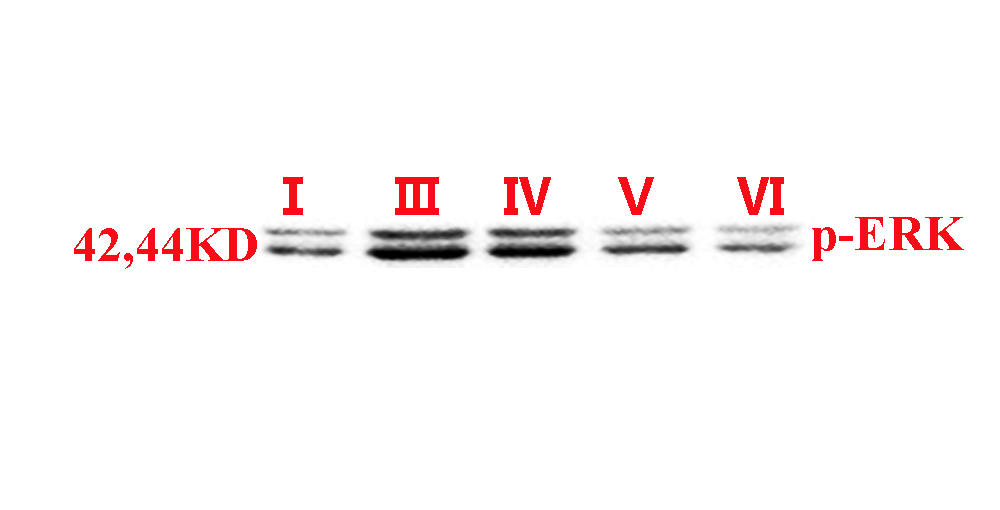
**

**
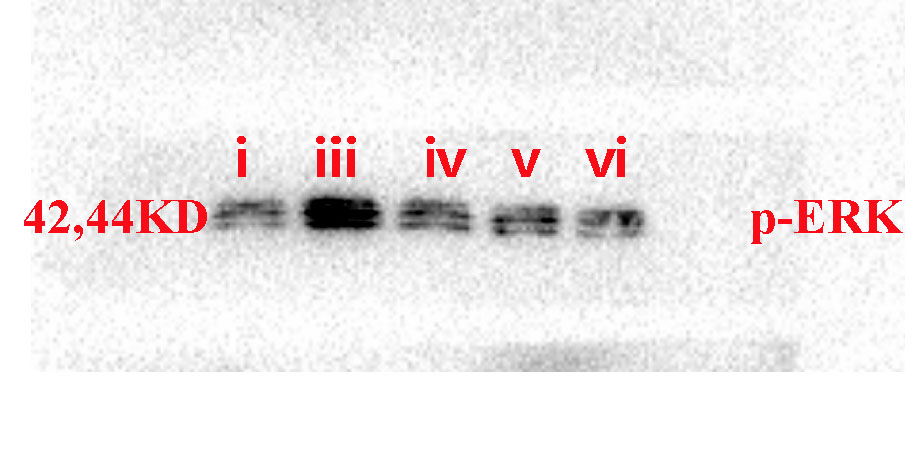
**

**
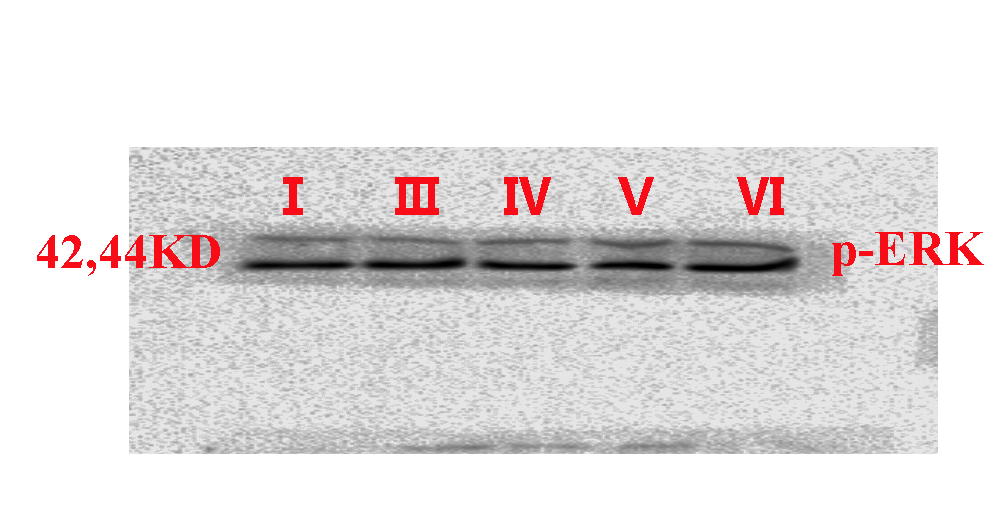
**

**
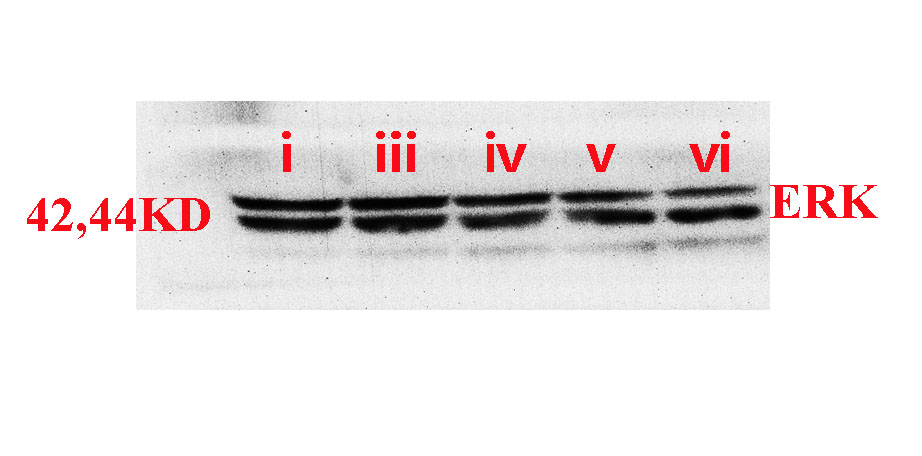
**

**
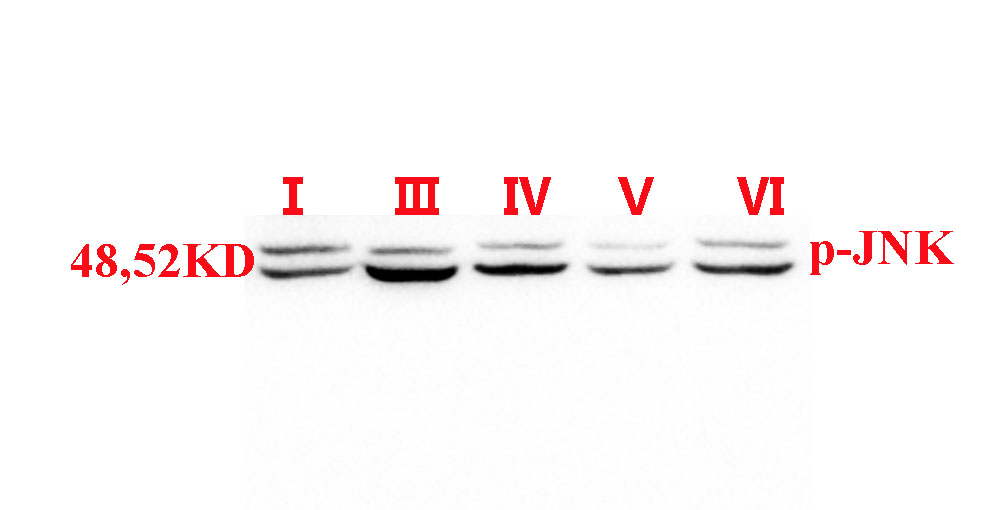
**

**
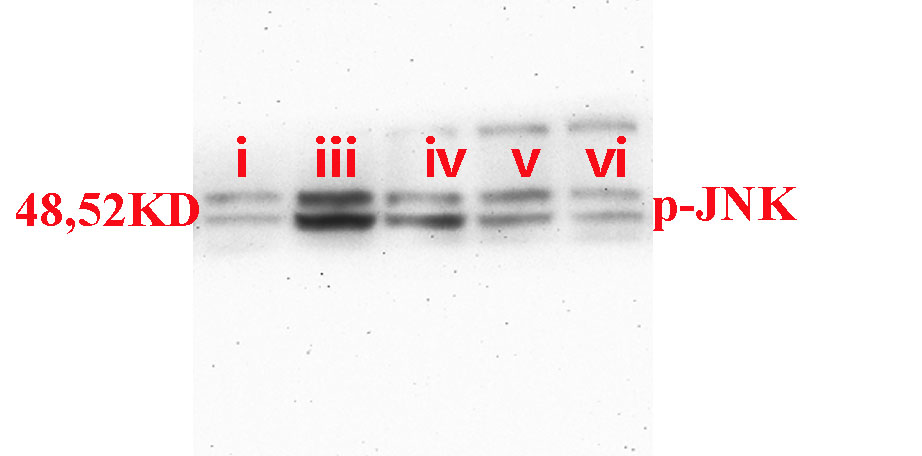
**

**
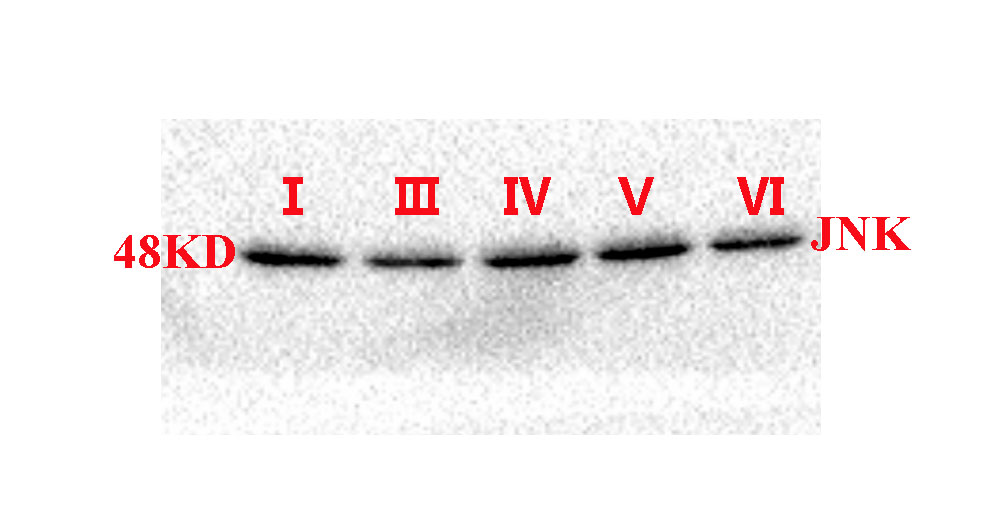
**

**
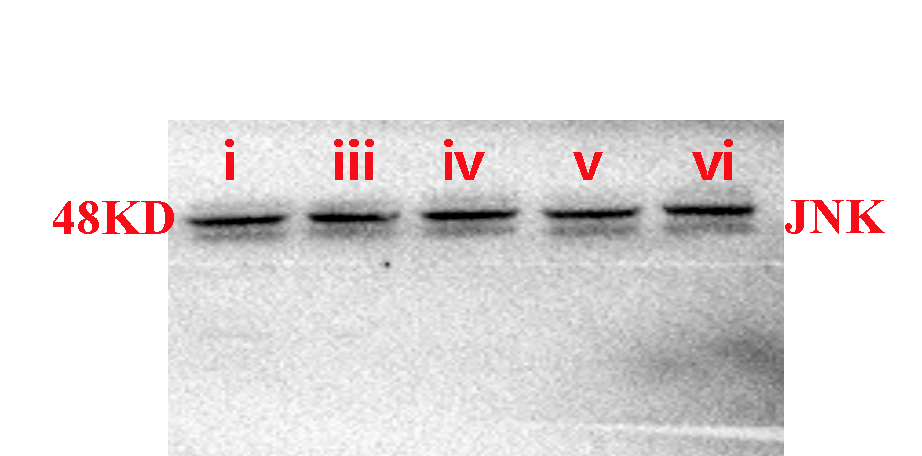
**

**
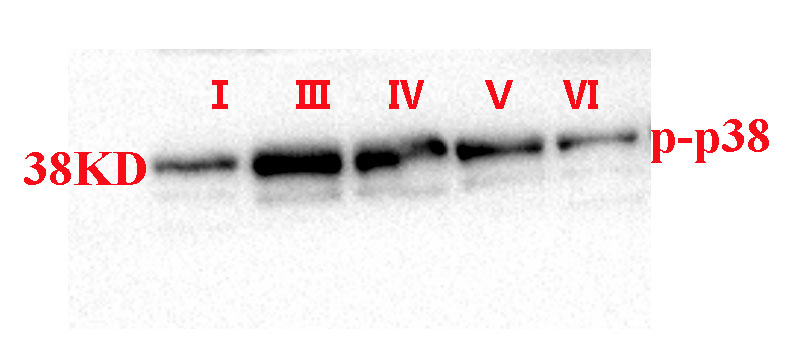
**

**
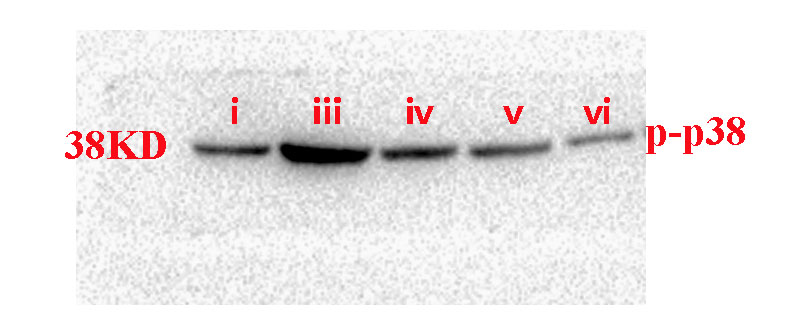
**

**
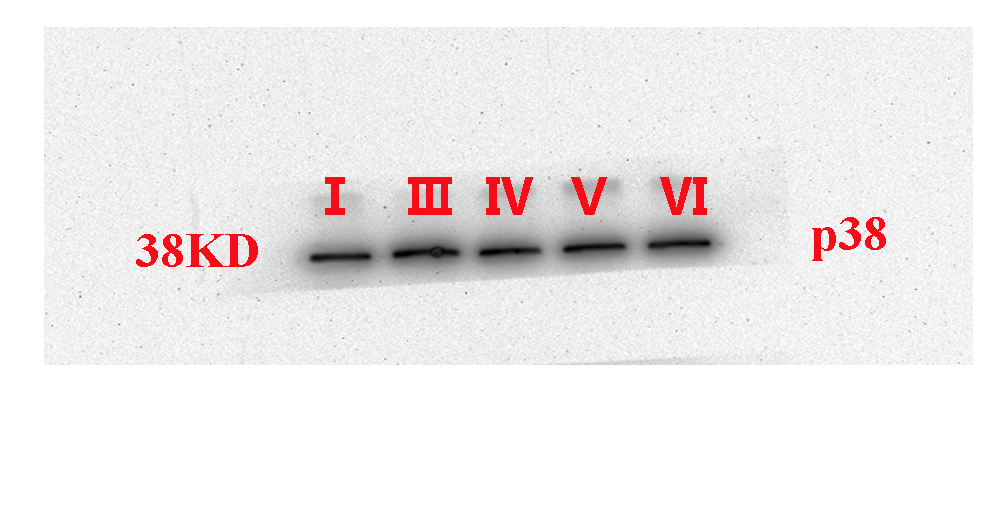
**

**
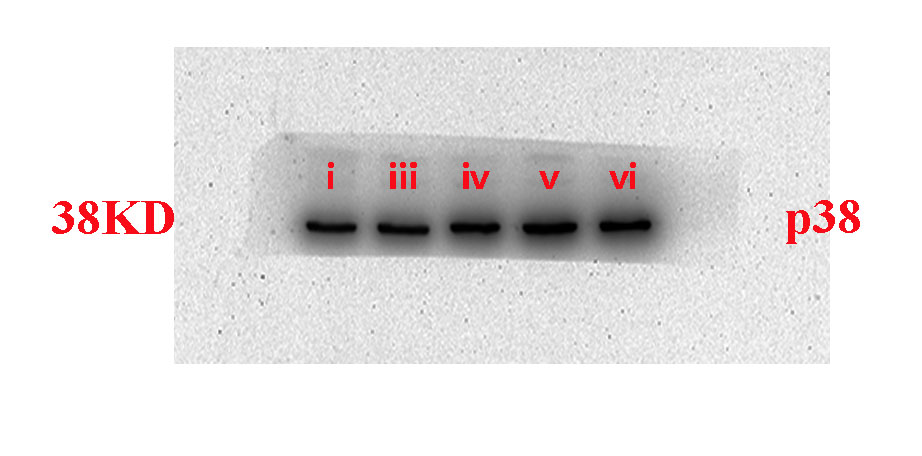
**

**
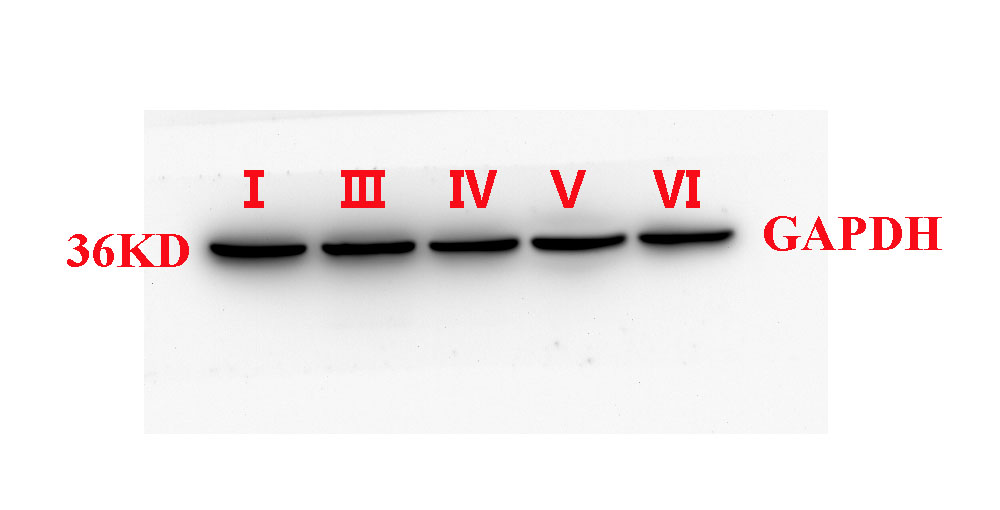
**

**
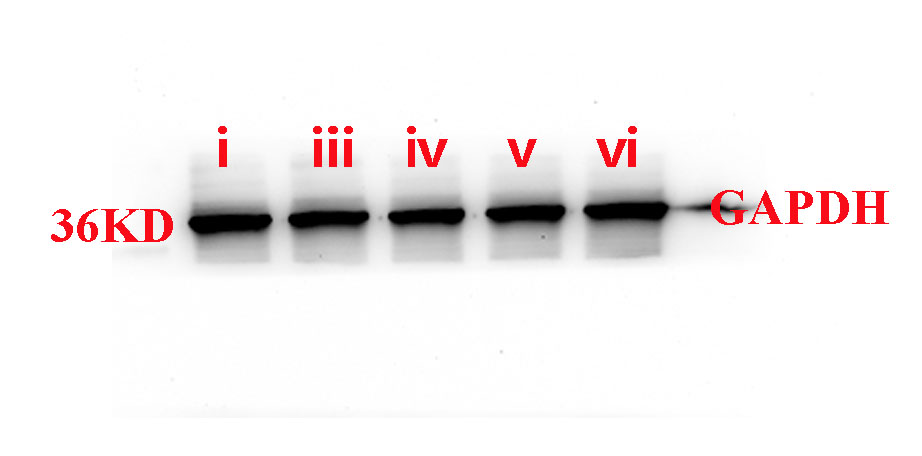
**

**Supplemental Figure 13.** Effects of dioscin on the protein expression levels of p-ERK, ERK, p-JNK, JNK, p-p38 and p38 *in vivo*.

**
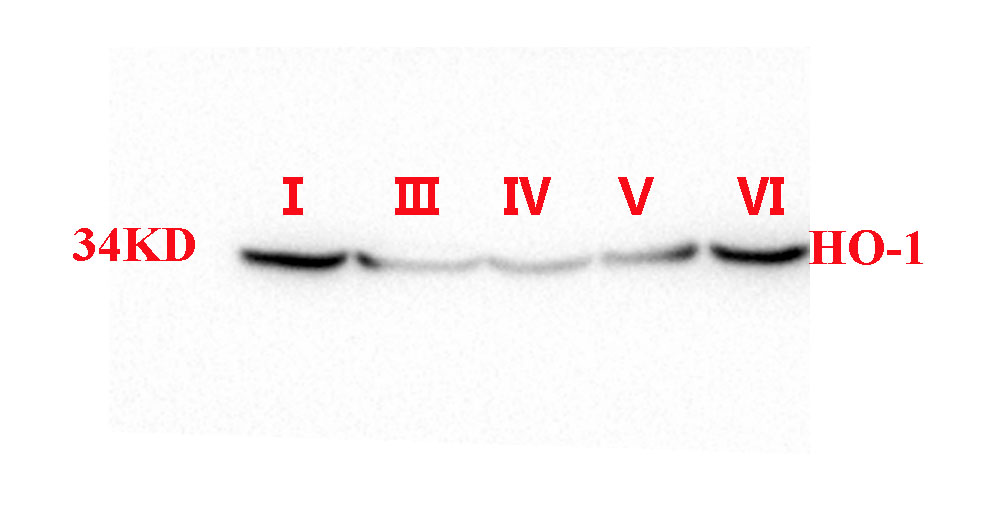
**

**
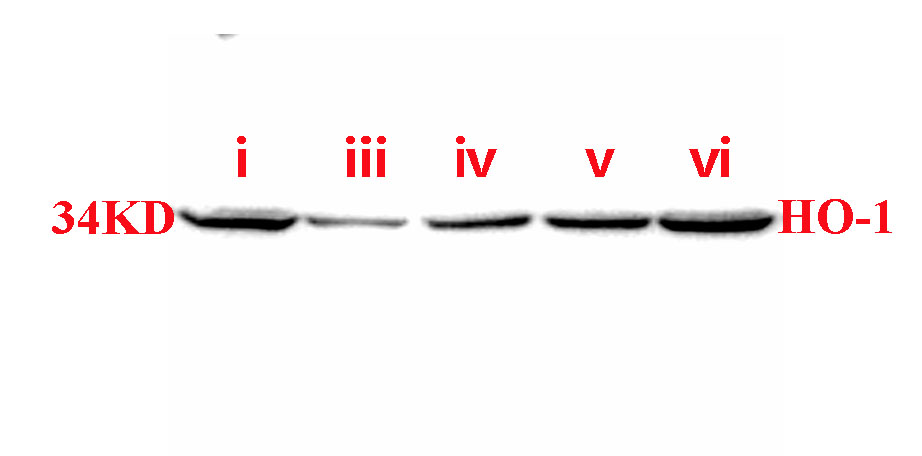
**

**
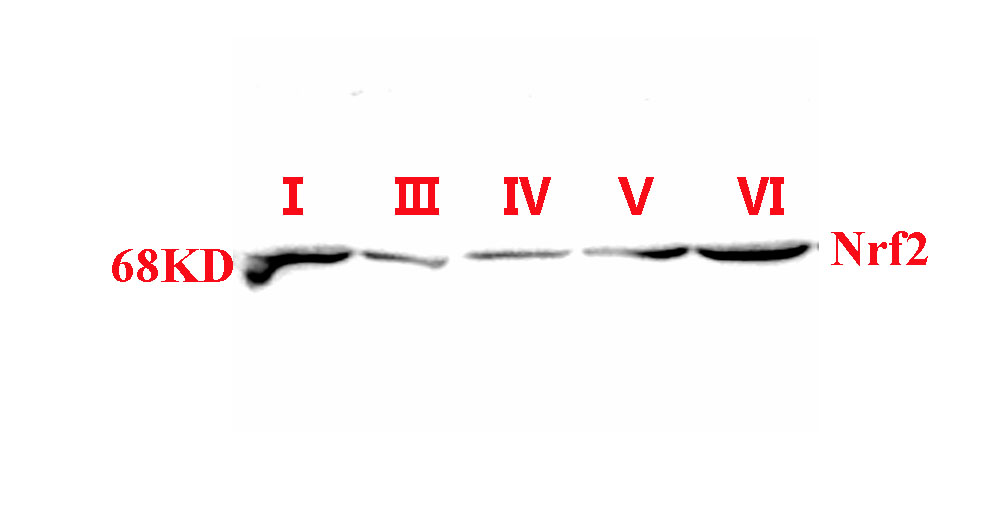
**

**
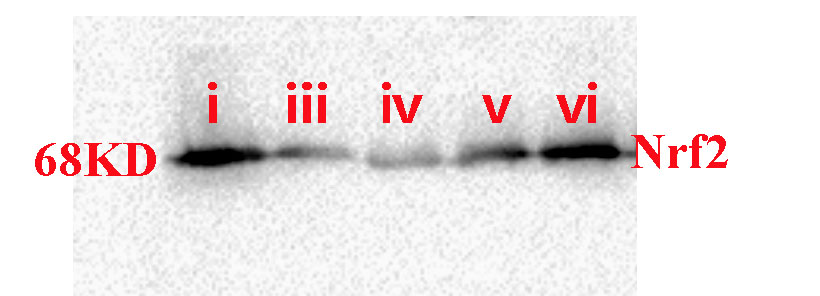
**

**
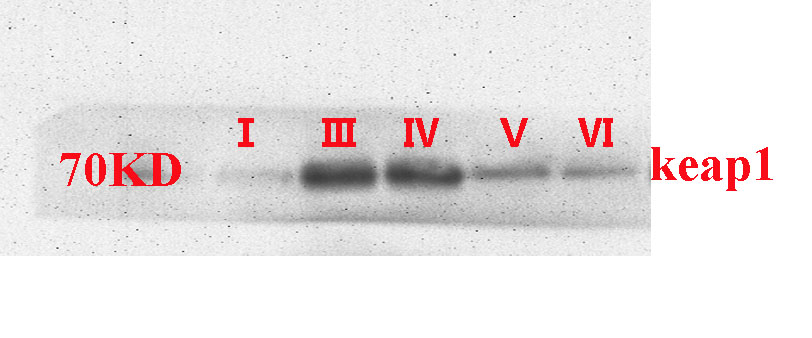
**

**
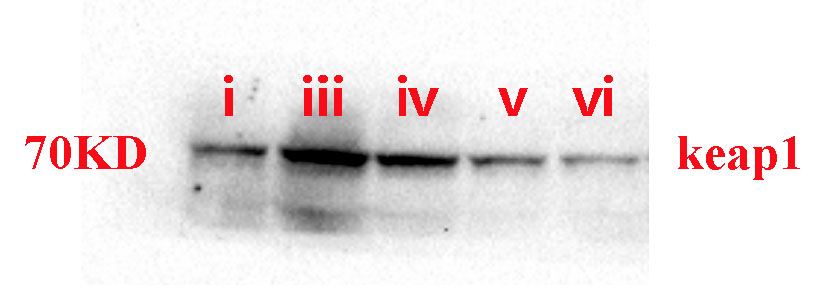
**

**
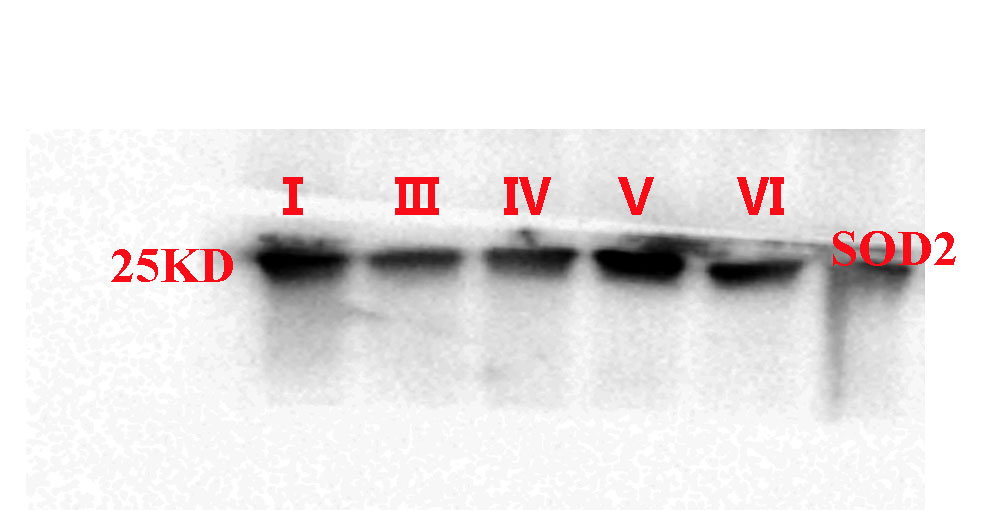
**

**
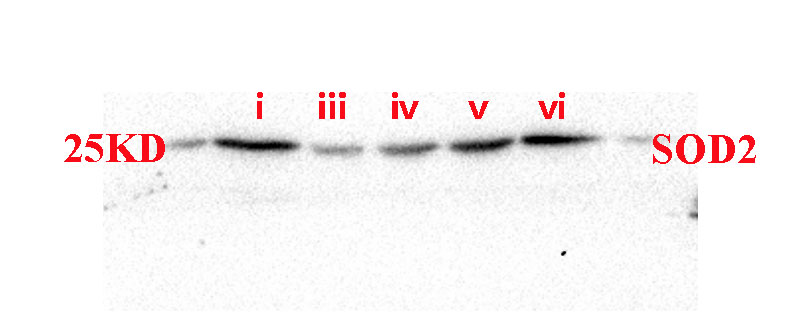
**

**
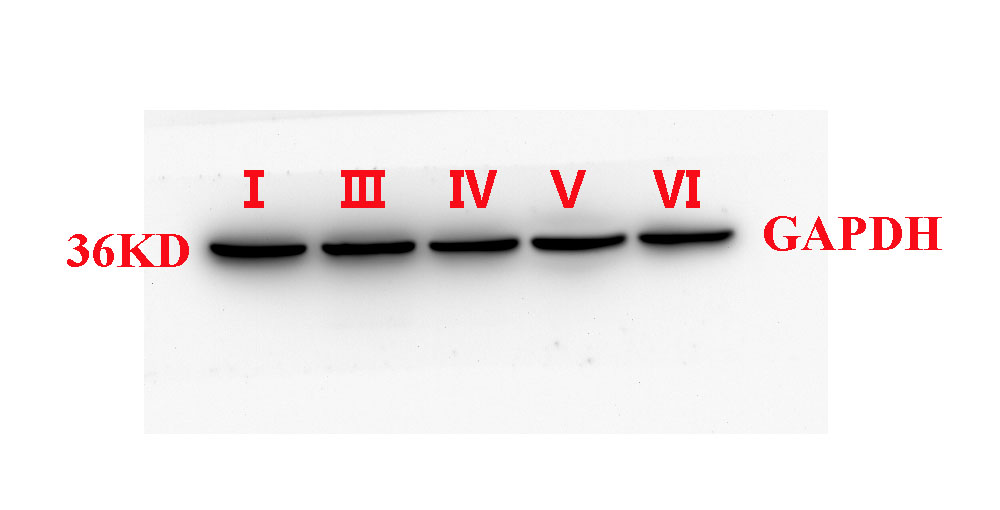
**

**
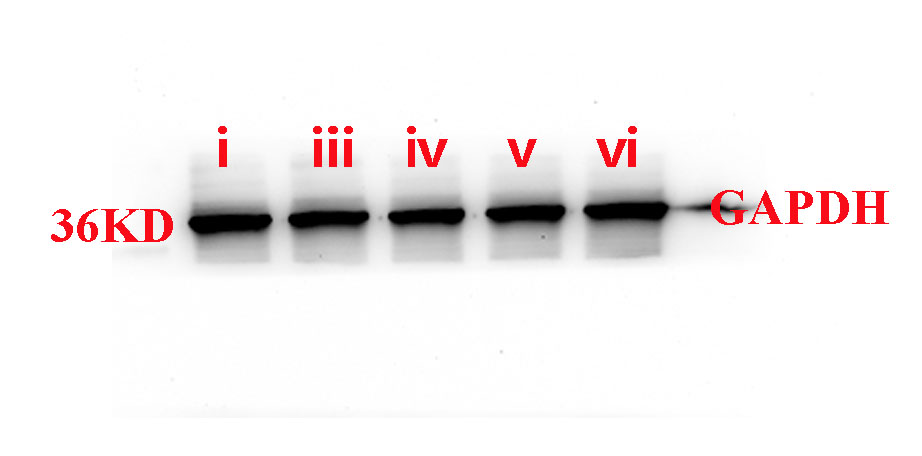
**

**Supplemental Figure 14.** Effects of dioscin on the protein expression levels of HO-1, Nrf2, keap1 and SOD2 *in vivo*.

**
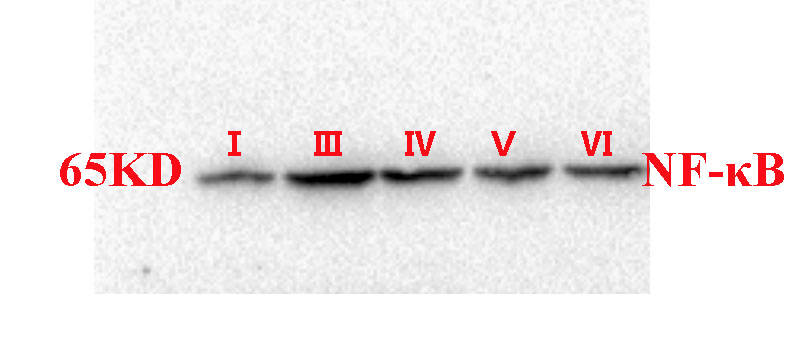
**

**
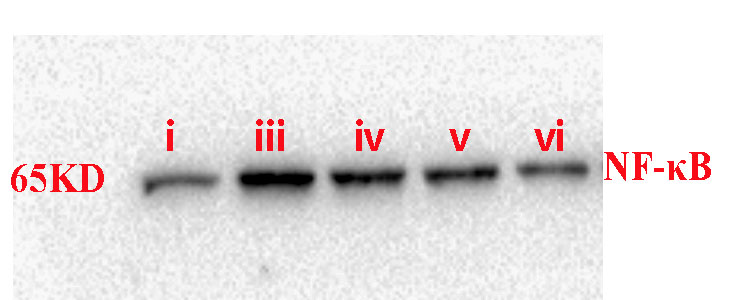
**

**
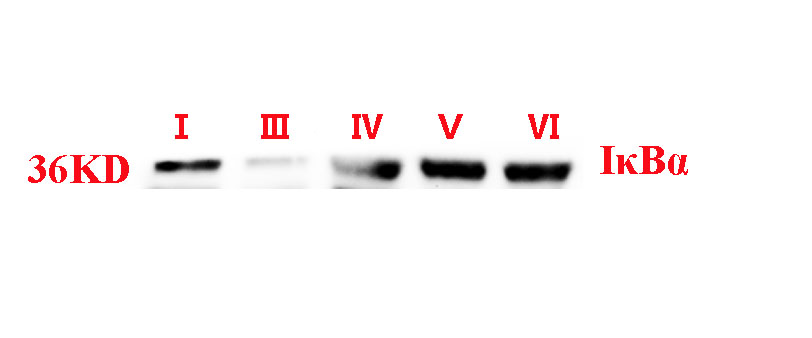
**

**
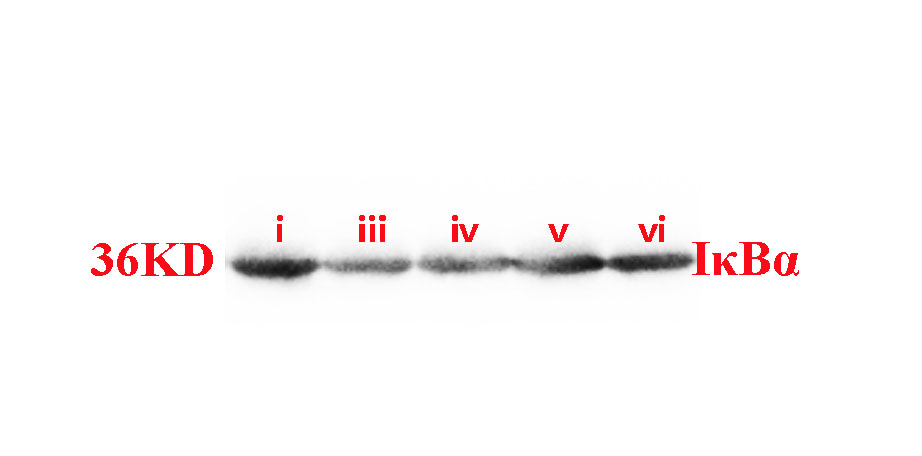
**

**
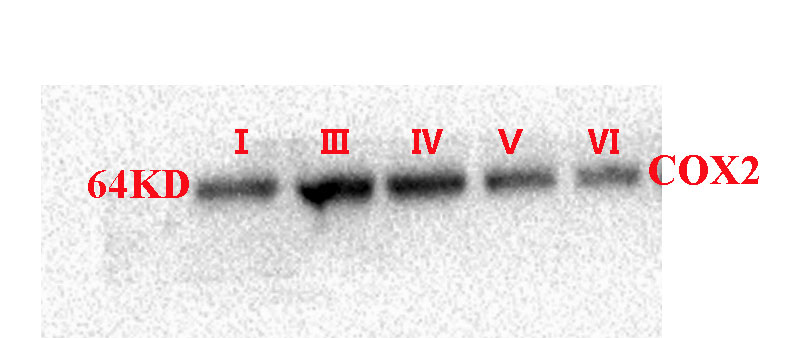
**

**
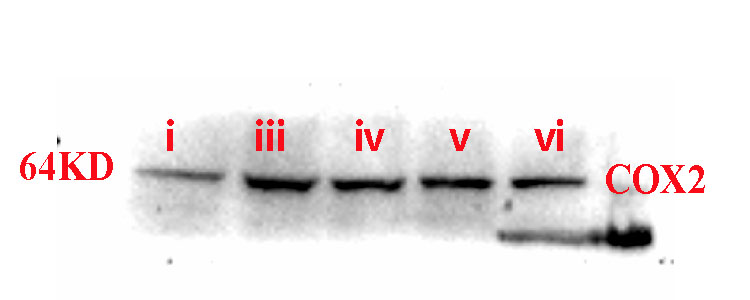
**

**
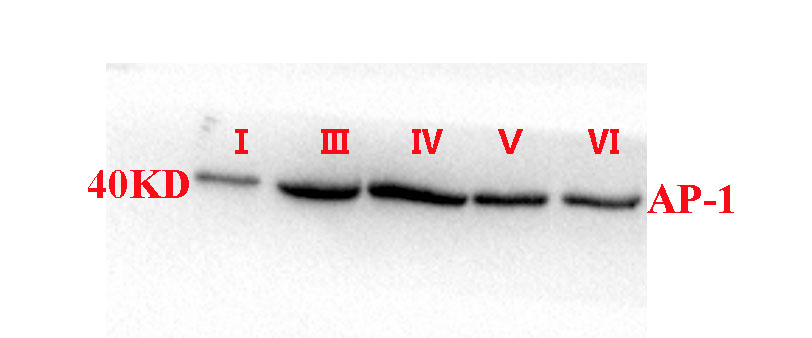
**

**
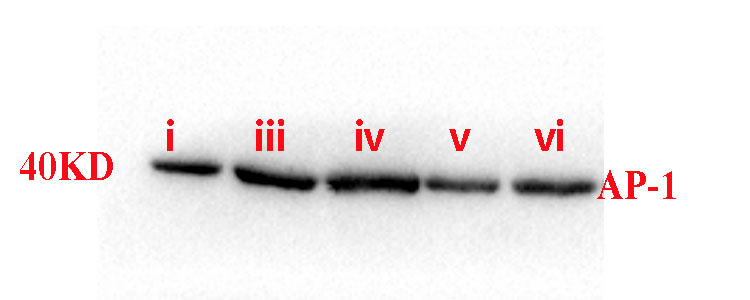
**

**
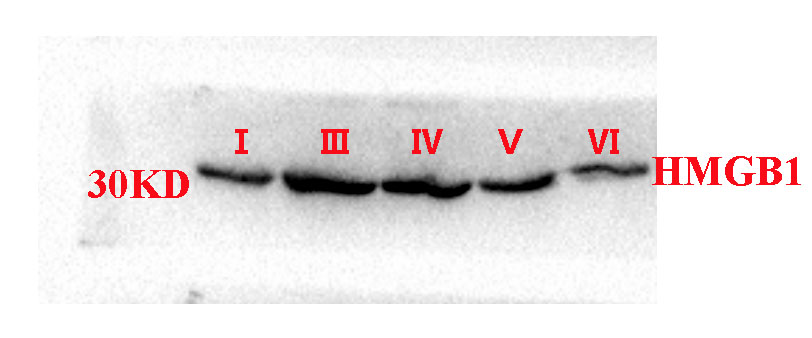
**

**
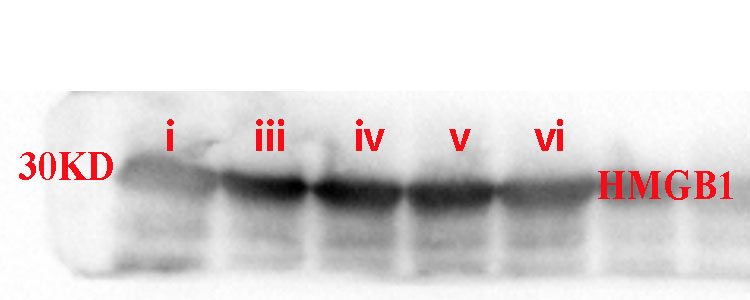
**

**
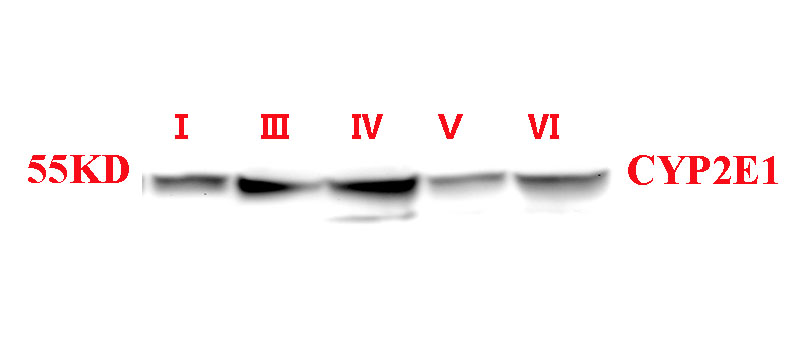
**

**
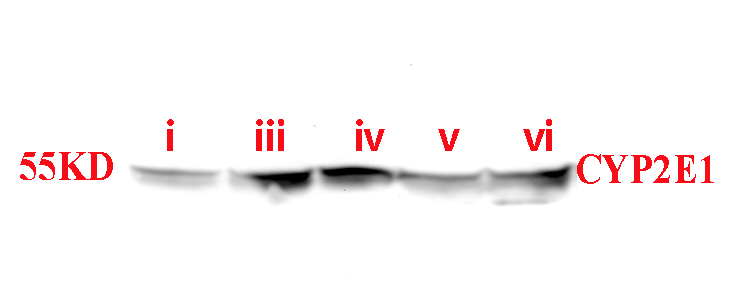
**

**Supplemental Figure 15.** Effects of dioscin on the protein expression levels of NF-κB, IκBα, COX2, AP-1, HMGB1 and CYP2E1 *in vivo*.

**Supplemental Figure 16.** Effects of dioscin on the protein expression level of p53 *in vivo*.

**Supplemental Figure 17.** Effects of dioscin on the protein expression levels of Cyt C, Bcl-2, Bcl-xl, BAX, BAK, Caspase 3 and Caspase 9 *in vivo*.
